# Supplementary figures and images for: Melanocyte differentiation and mechanosensation are differentially modulated by distinct extracellular matrix proteins
Source: EMBO Rep. 2025 Sep 19;26(21):5270–99. doi: 10.1038/s44319-025-00583-6 (PMC12592508; doi:10.1038/s44319-025-00583-6)

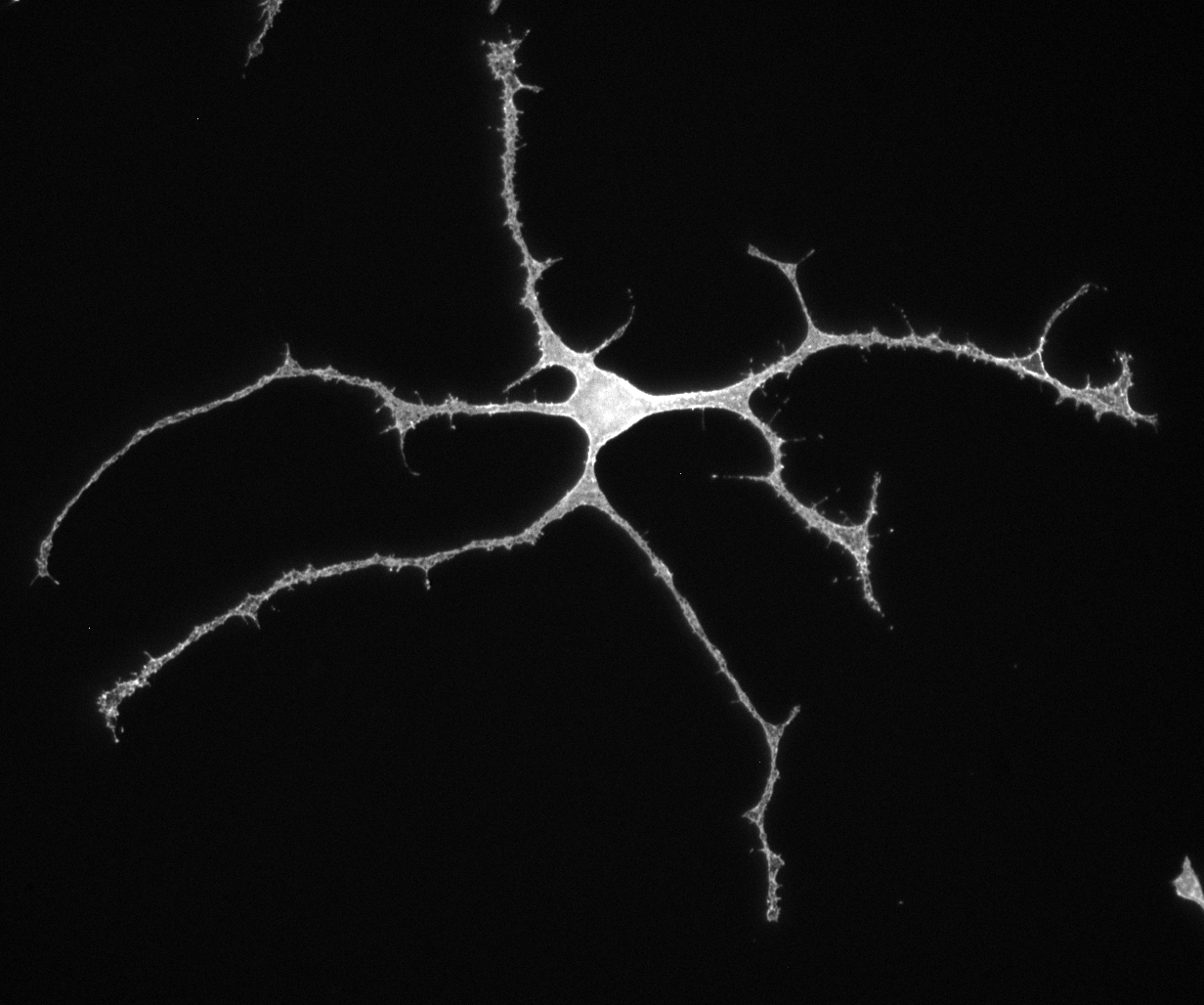

Supplement: Supplementary file 2 — Source data Fig. 1 [file 44319_2025_583_MOESM2_ESM.zip › Figure 1/1A/1A_td-Tomato_1-10_FN.tif]

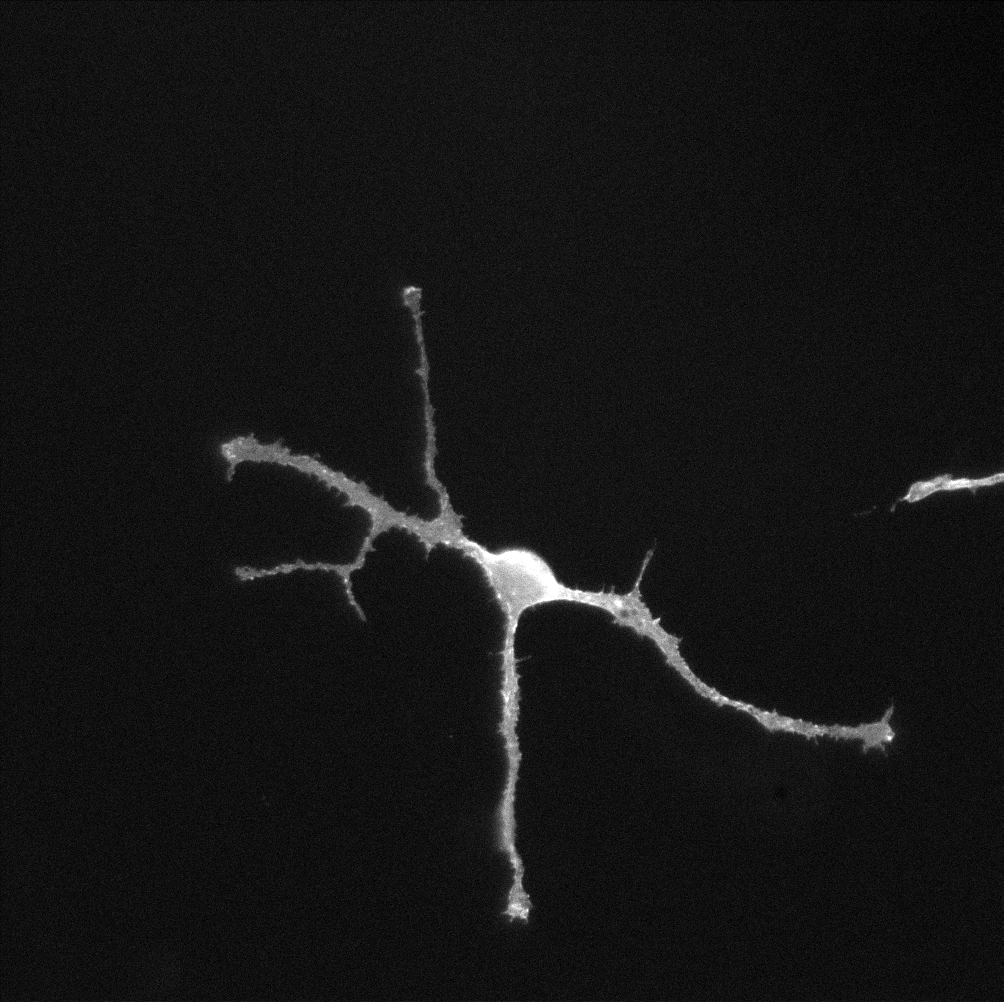

Supplement: Supplementary file 2 — Source data Fig. 1 [file 44319_2025_583_MOESM2_ESM.zip › Figure 1/1A/1A_td-Tomato_1-10_COLIV.tif]

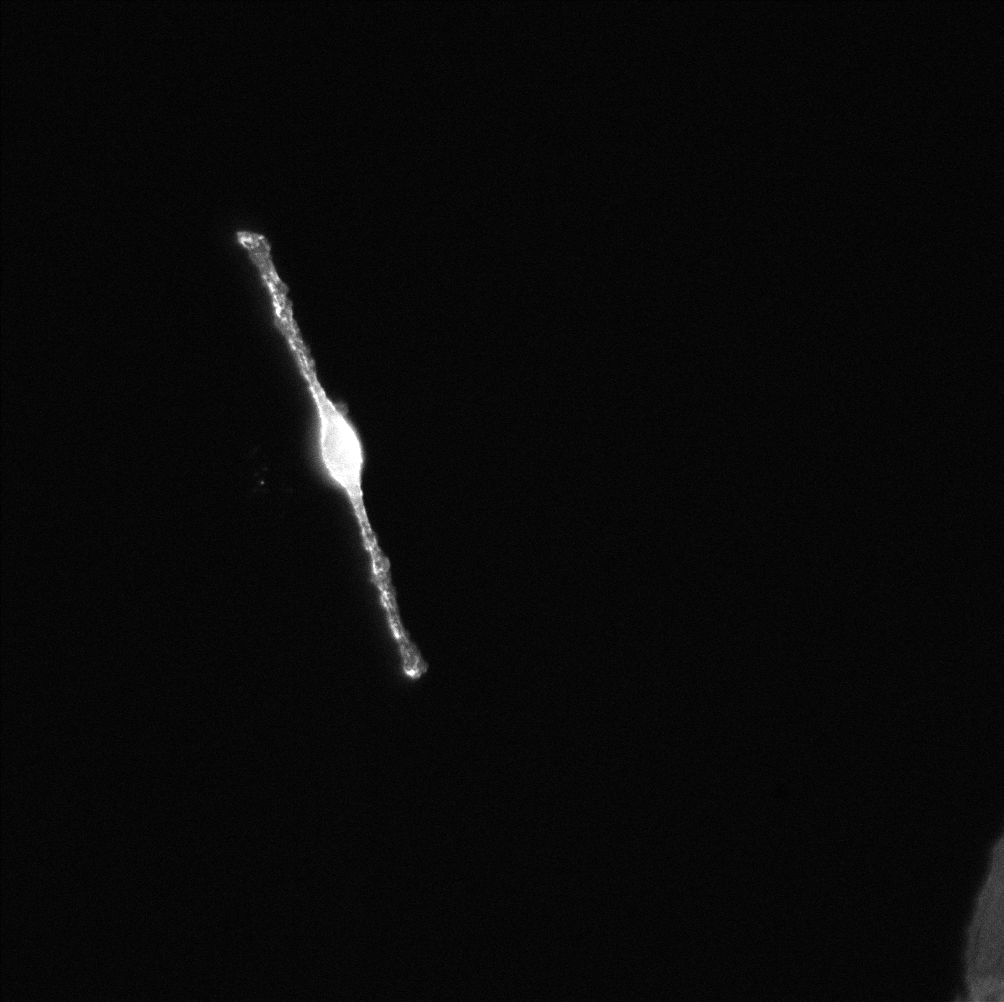

Supplement: Supplementary file 2 — Source data Fig. 1 [file 44319_2025_583_MOESM2_ESM.zip › Figure 1/1A/1A_td-Tomato_1-10_COLI.tif]

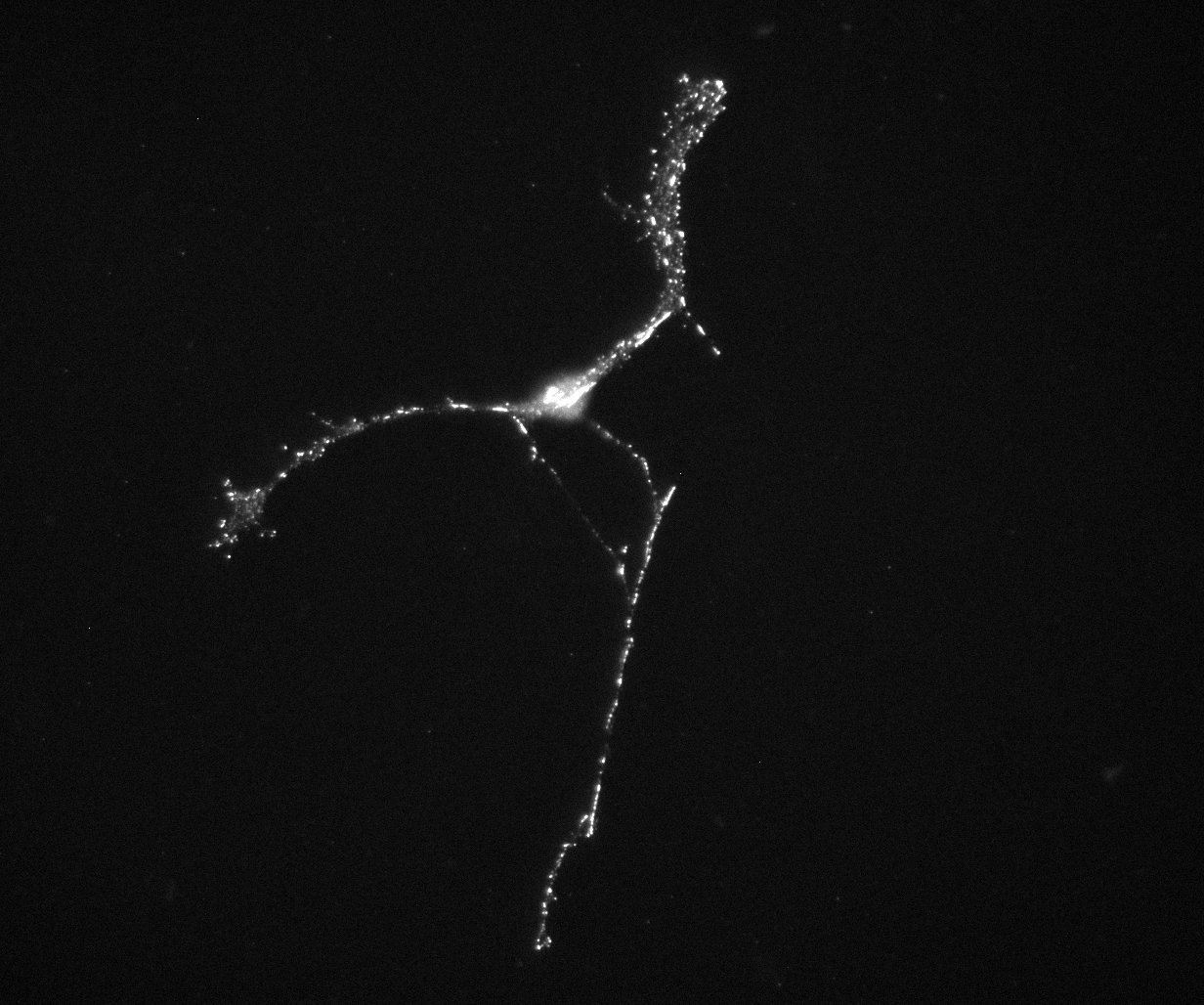

Supplement: Supplementary file 2 — Source data Fig. 1 [file 44319_2025_583_MOESM2_ESM.zip › Figure 1/1D/1B_COLIV_1-10_pFAK.tif]

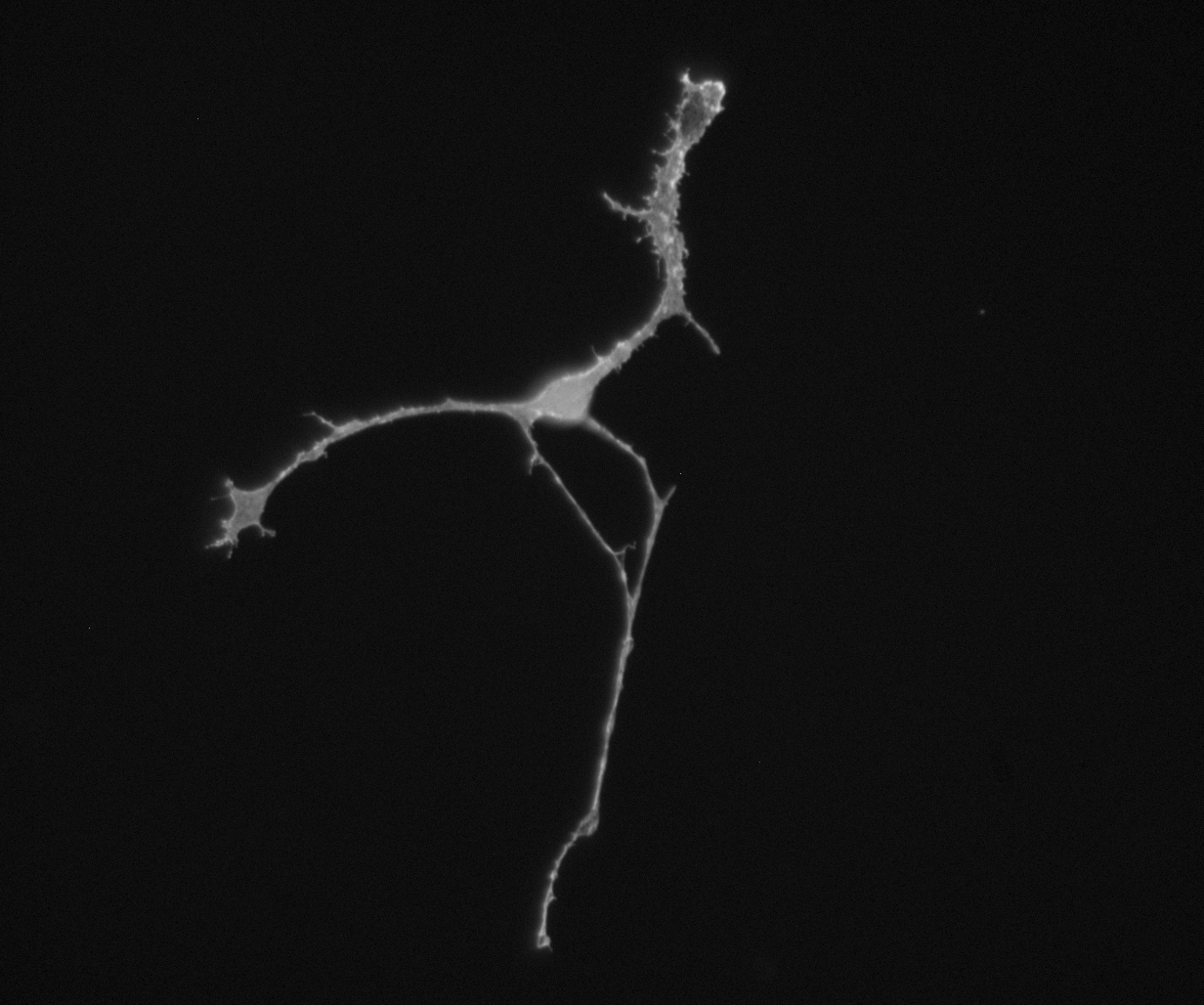

Supplement: Supplementary file 2 — Source data Fig. 1 [file 44319_2025_583_MOESM2_ESM.zip › Figure 1/1D/1B_COLIV_1-10_td-Tomato.tif]

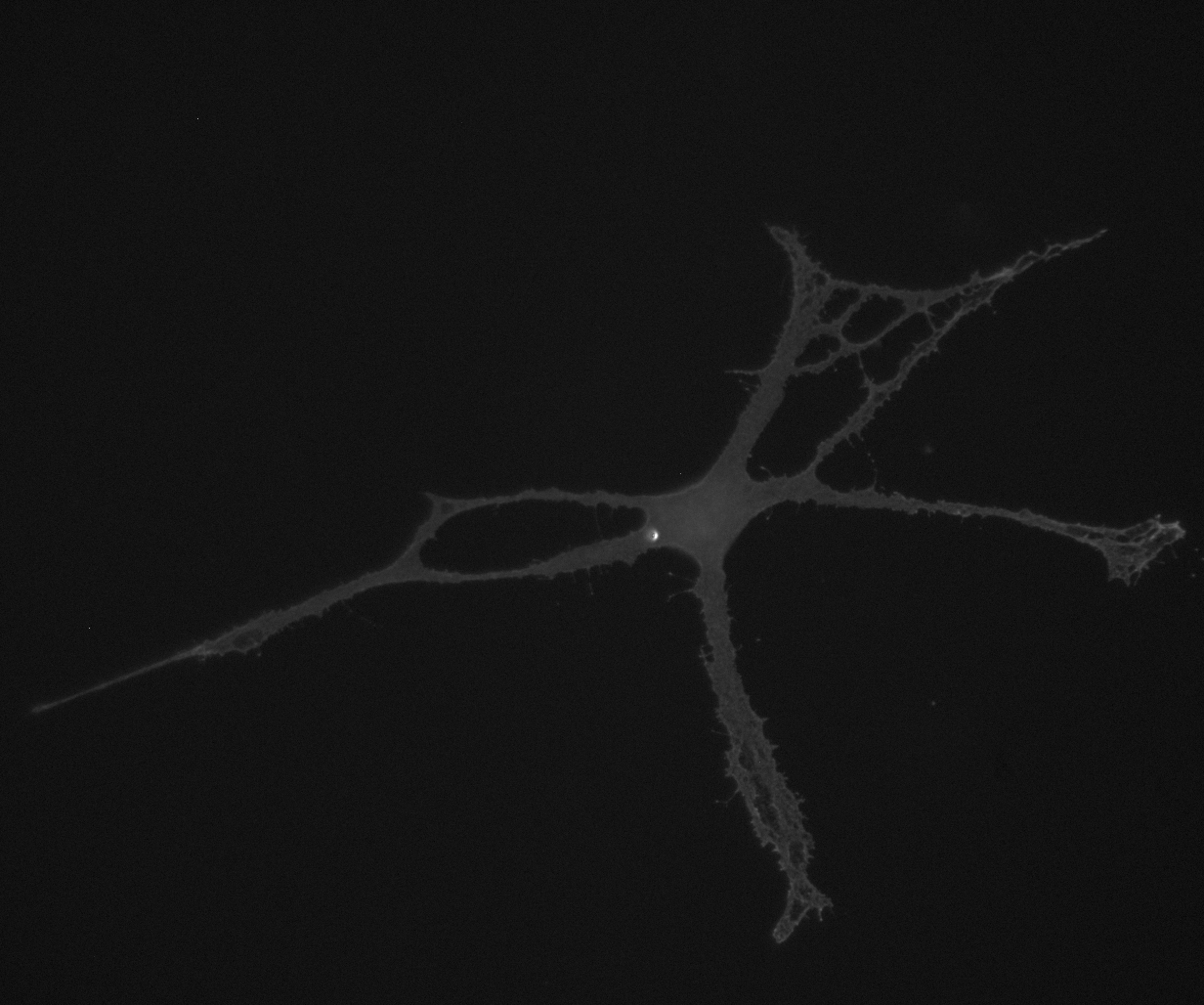

Supplement: Supplementary file 2 — Source data Fig. 1 [file 44319_2025_583_MOESM2_ESM.zip › Figure 1/1D/1B_FN_1-10_td-Tomato.tif]

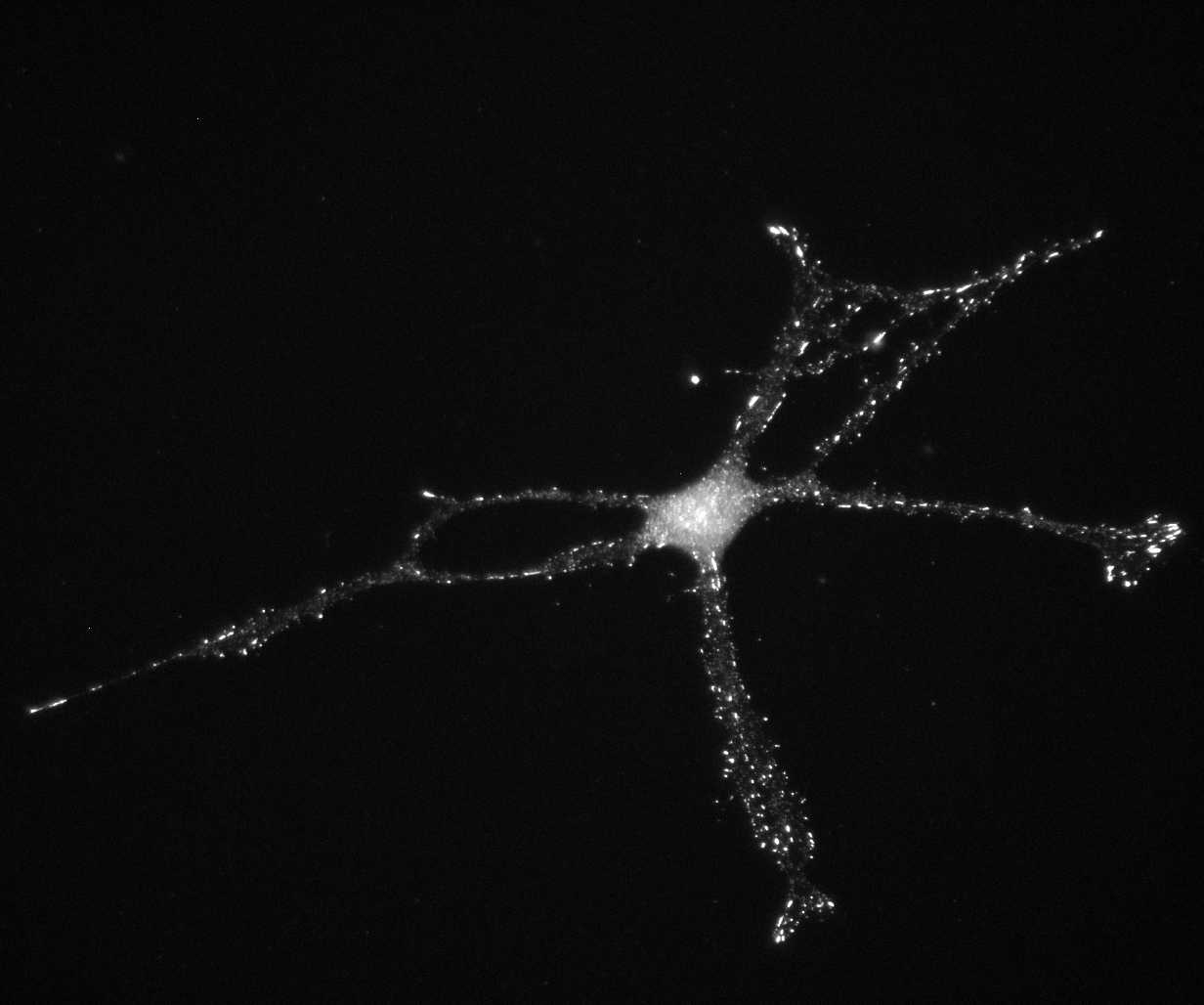

Supplement: Supplementary file 2 — Source data Fig. 1 [file 44319_2025_583_MOESM2_ESM.zip › Figure 1/1D/1B_FN_1-10_pFAK.tif]

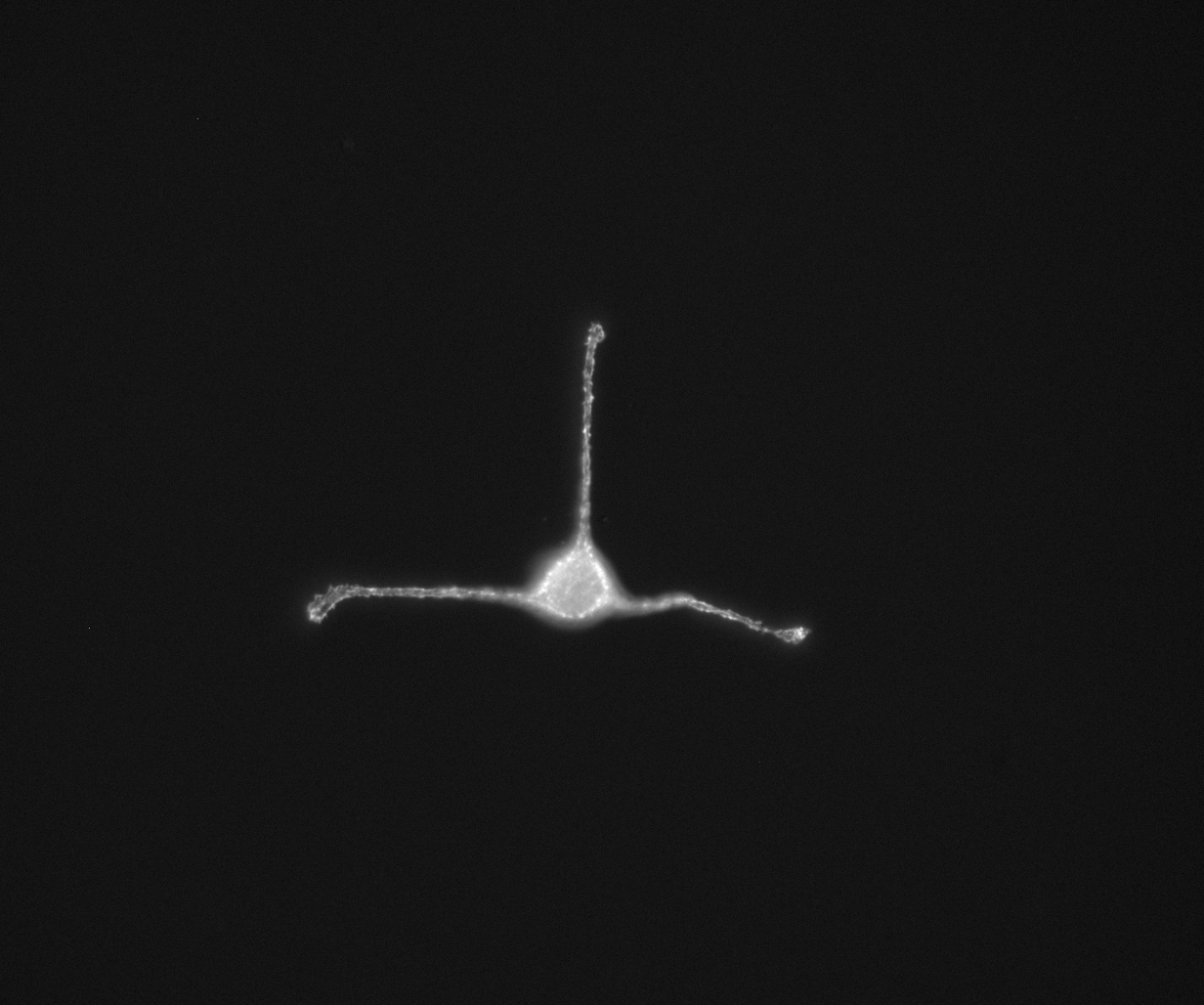

Supplement: Supplementary file 2 — Source data Fig. 1 [file 44319_2025_583_MOESM2_ESM.zip › Figure 1/1D/1B_COLI_1-10_td-Tomato.tif]

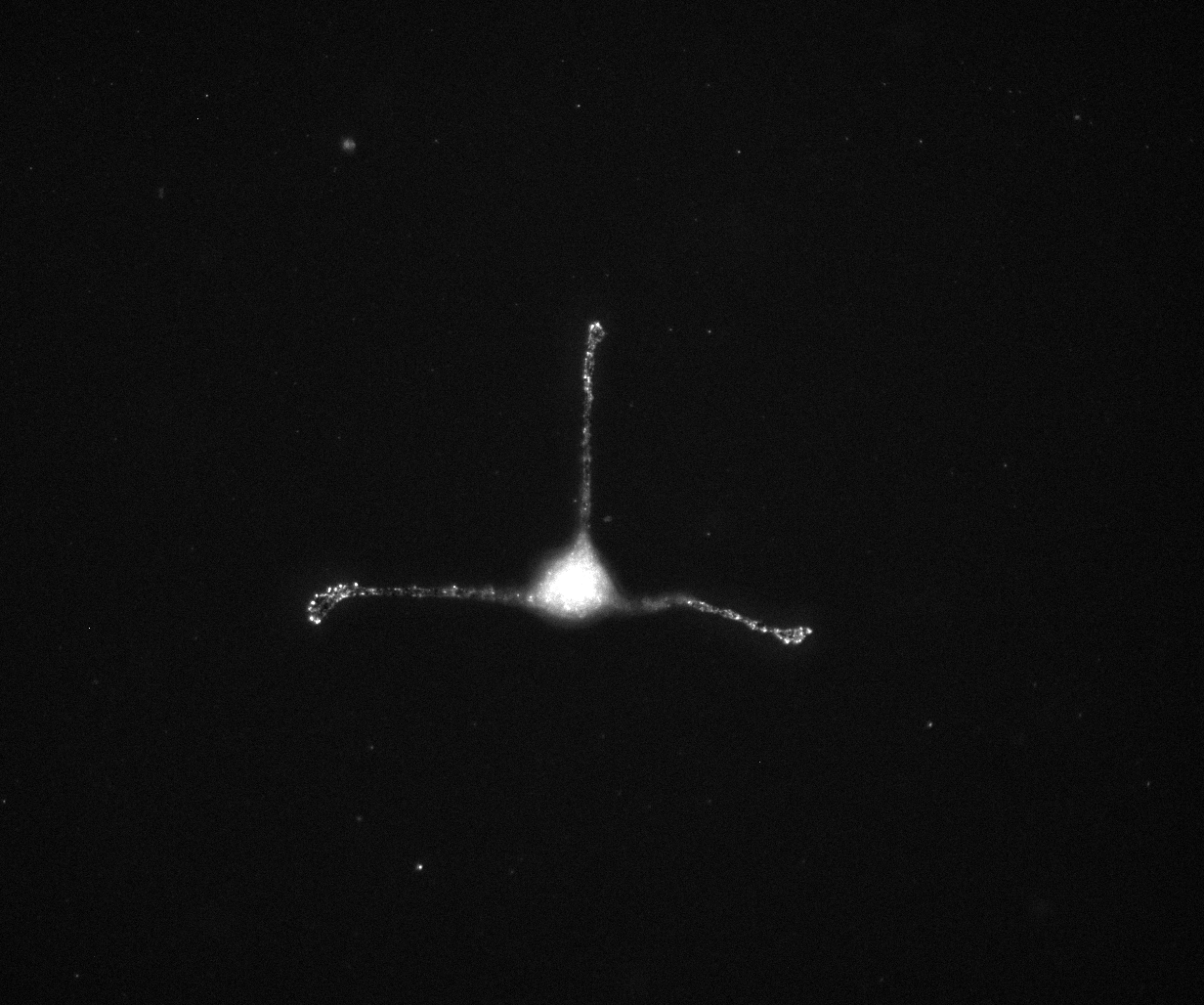

Supplement: Supplementary file 2 — Source data Fig. 1 [file 44319_2025_583_MOESM2_ESM.zip › Figure 1/1D/1B_COLI_1-10_pFAK.tif]

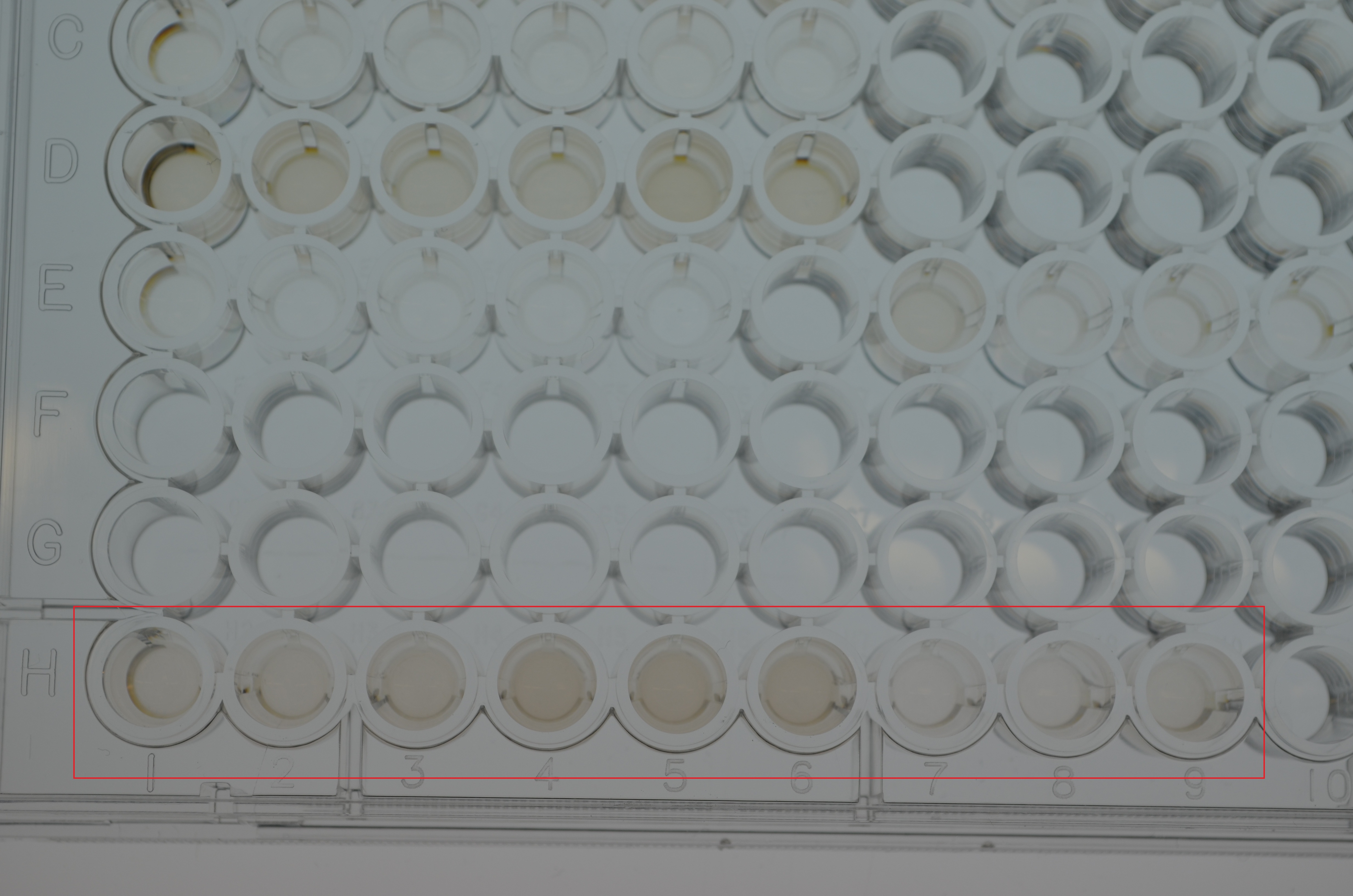

Supplement: Supplementary file 4 — Source data Fig. 3 [file 44319_2025_583_MOESM4_ESM.zip › Figure 3/3A/figure3A.jpg]

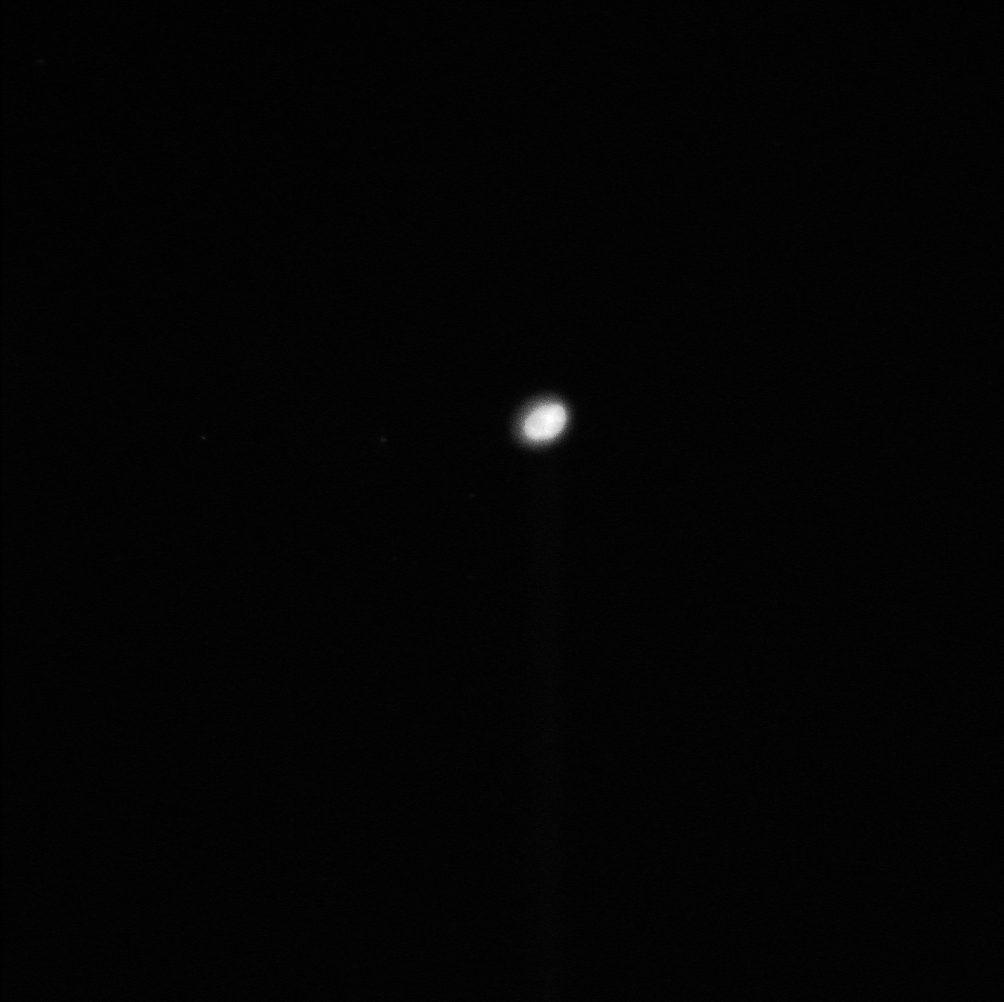

Supplement: Supplementary file 5 — Source data Fig. 4 [file 44319_2025_583_MOESM5_ESM.zip › Figure 4/4A/4A_COLI_1-10_dapi.tif]

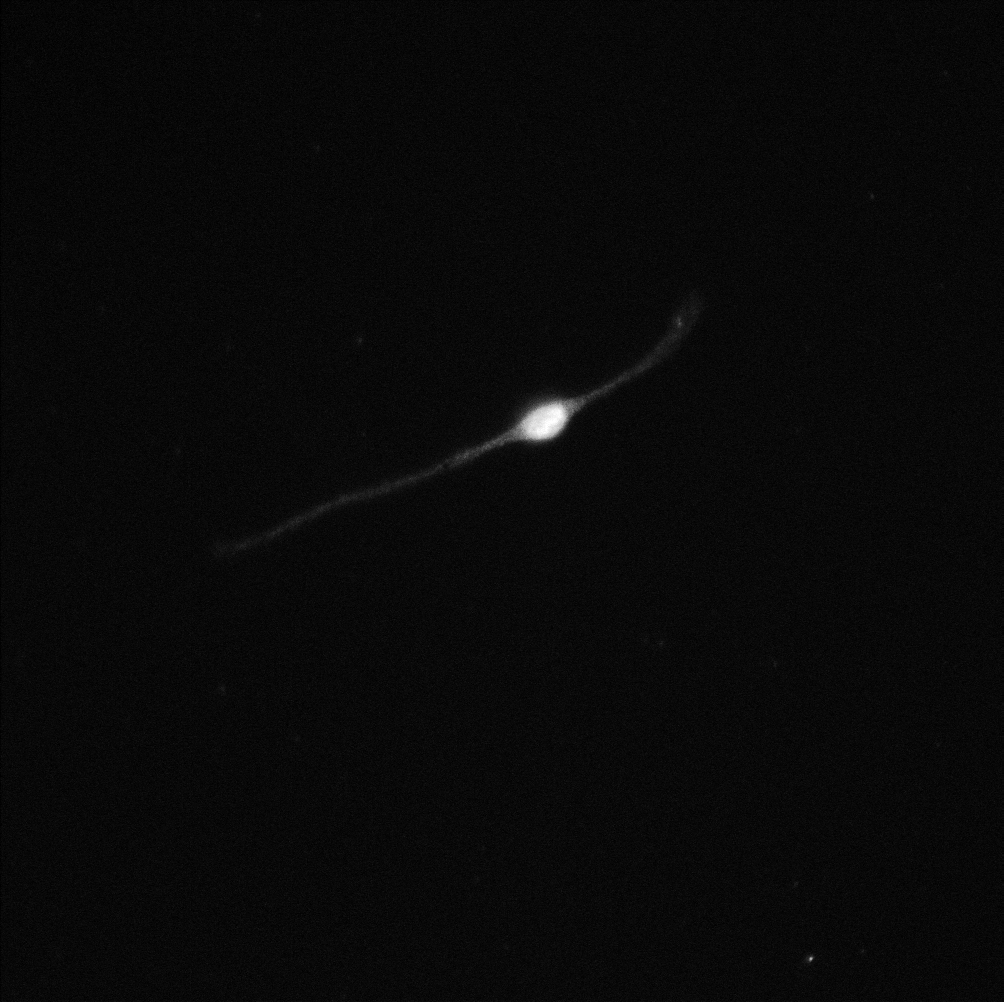

Supplement: Supplementary file 5 — Source data Fig. 4 [file 44319_2025_583_MOESM5_ESM.zip › Figure 4/4A/4A_COLI_1-10_MITF.tif]

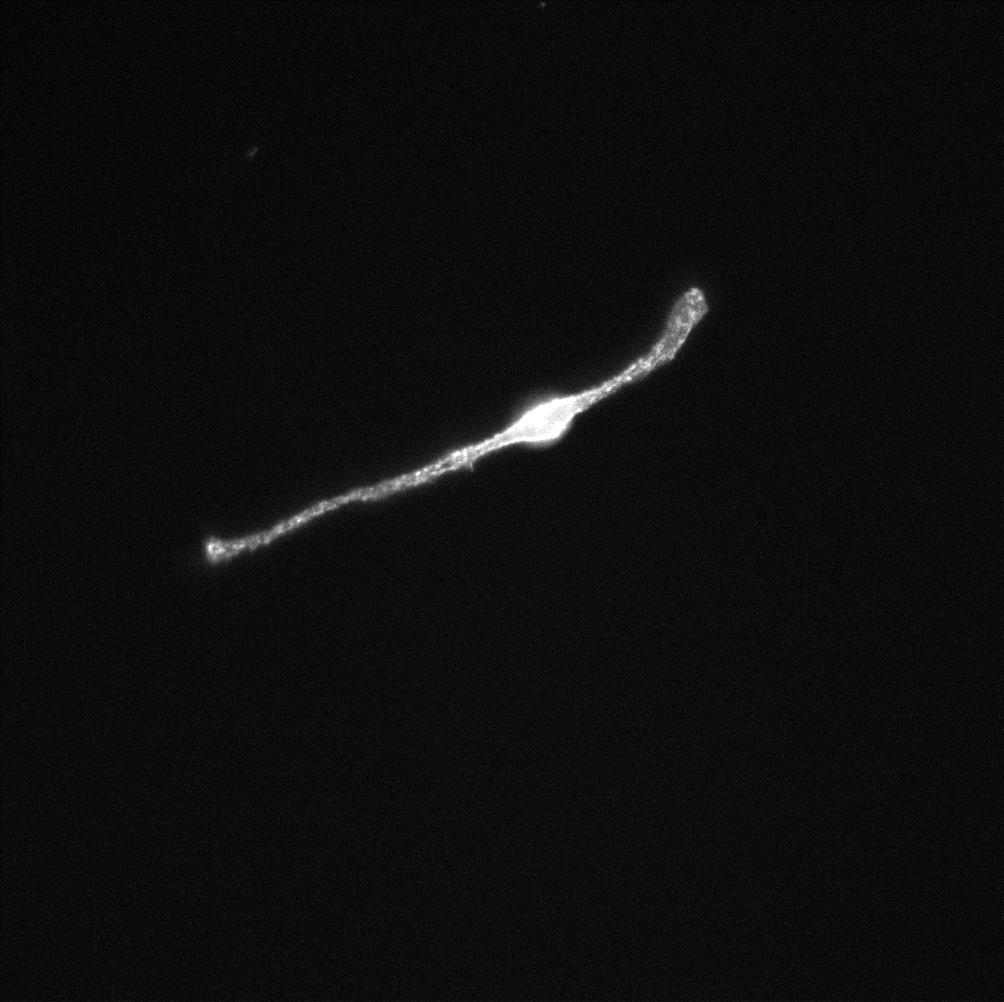

Supplement: Supplementary file 5 — Source data Fig. 4 [file 44319_2025_583_MOESM5_ESM.zip › Figure 4/4A/4A_COLI_1-10_td-Tomato.tif]

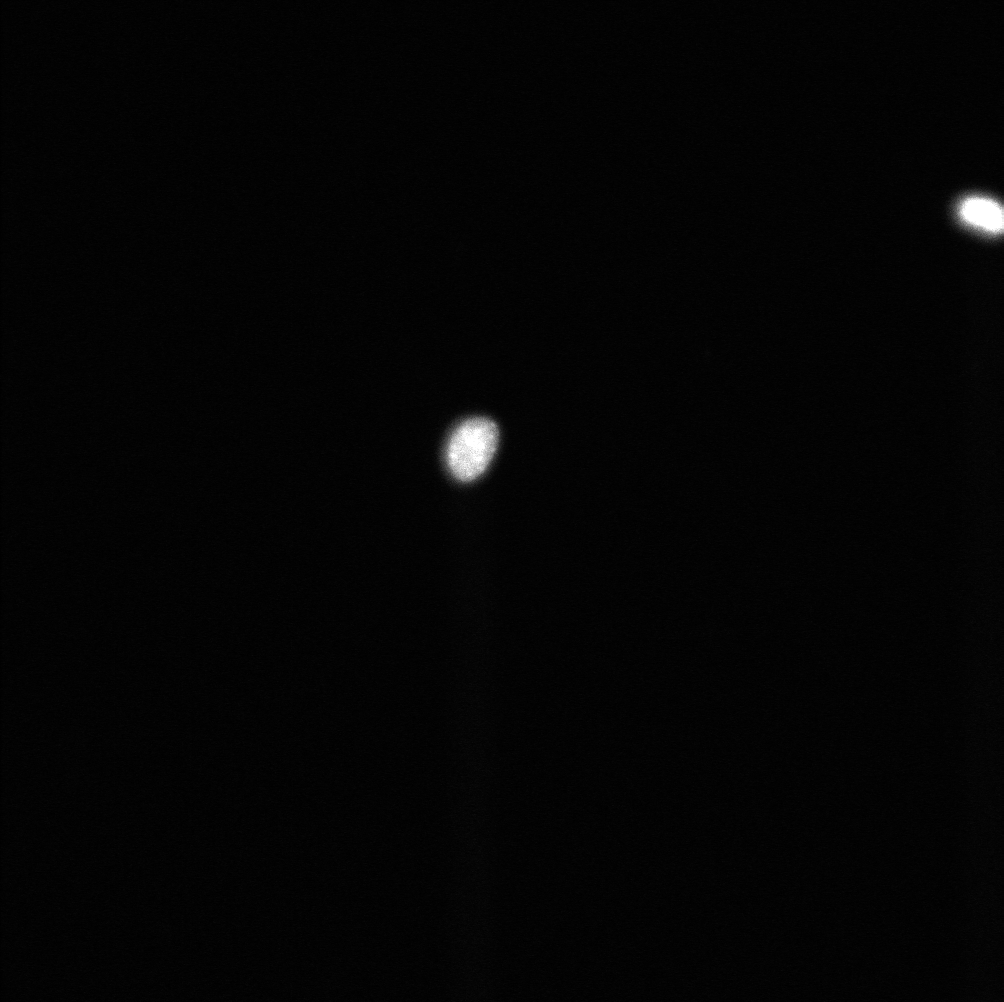

Supplement: Supplementary file 5 — Source data Fig. 4 [file 44319_2025_583_MOESM5_ESM.zip › Figure 4/4A/4A_FN_1-10_dapi.tif]

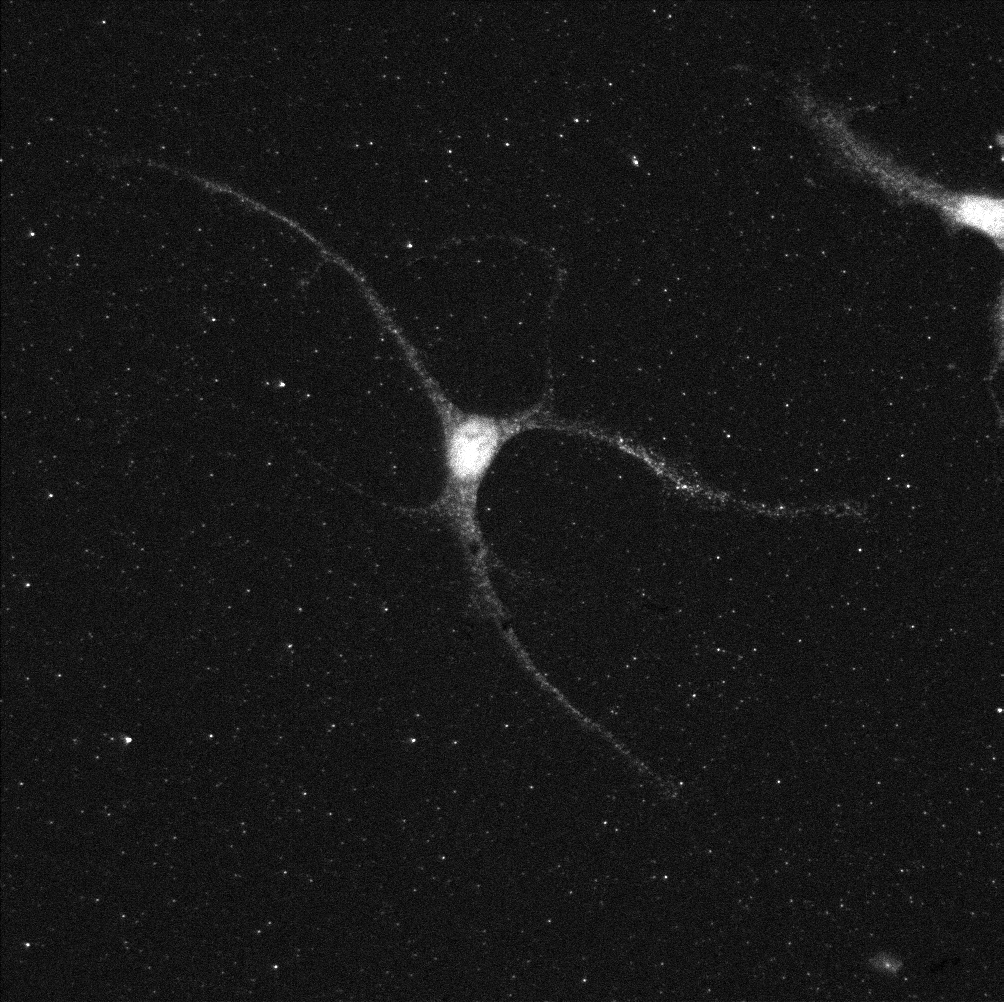

Supplement: Supplementary file 5 — Source data Fig. 4 [file 44319_2025_583_MOESM5_ESM.zip › Figure 4/4A/4A_FN_1-10_MITF.tif]

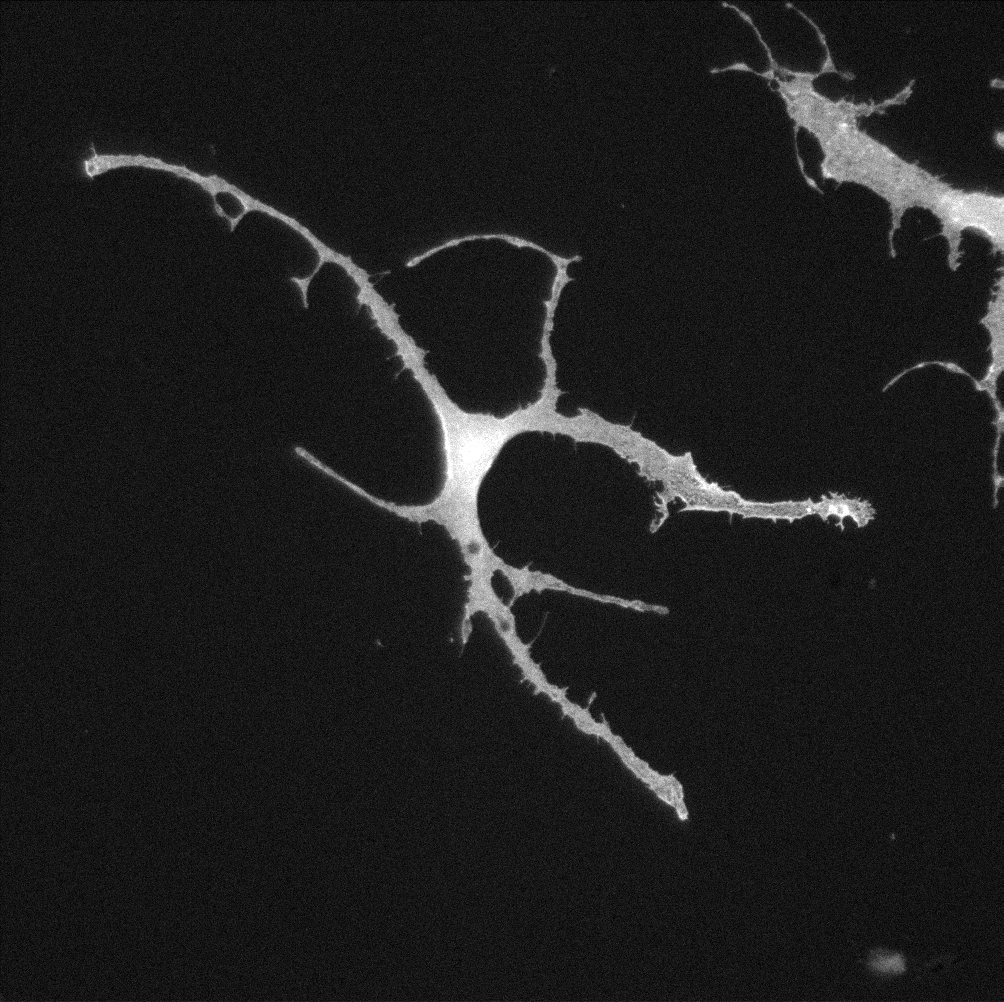

Supplement: Supplementary file 5 — Source data Fig. 4 [file 44319_2025_583_MOESM5_ESM.zip › Figure 4/4A/4A_FN_1-10_td-Tomato.tif]

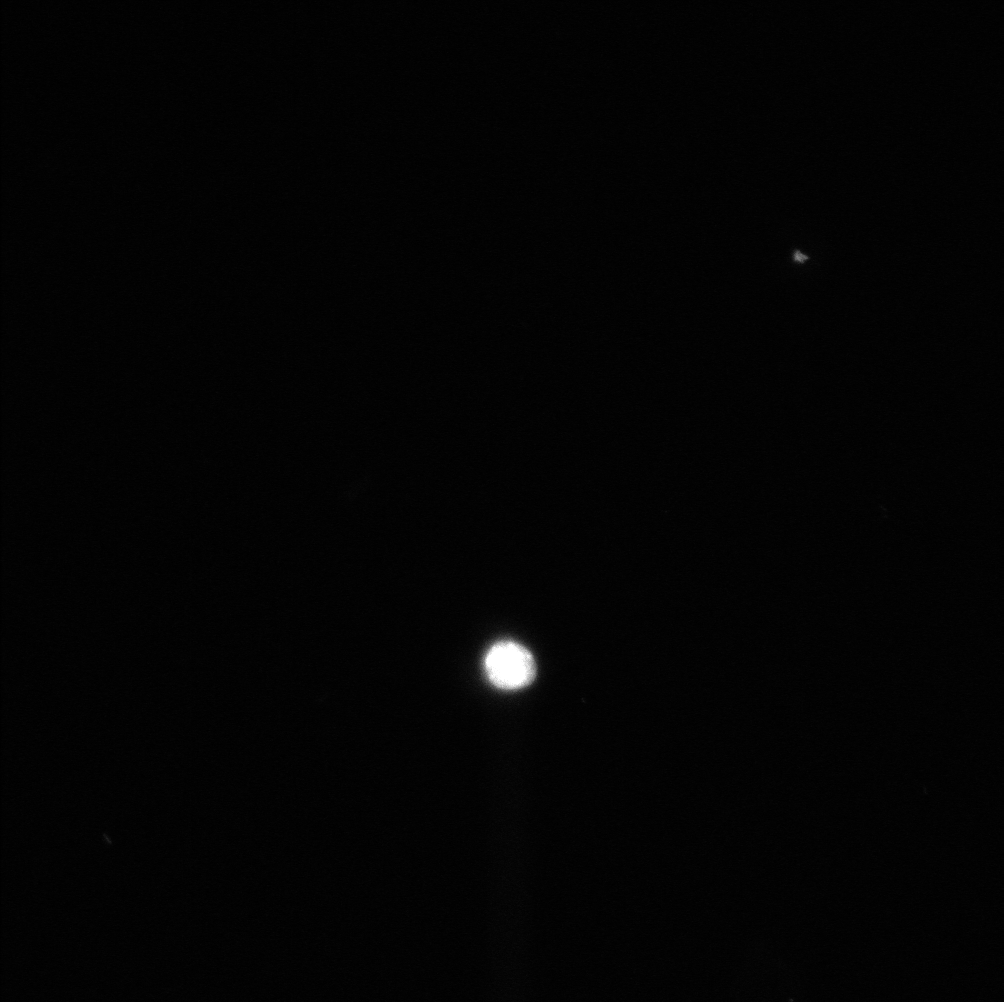

Supplement: Supplementary file 5 — Source data Fig. 4 [file 44319_2025_583_MOESM5_ESM.zip › Figure 4/4A/4A_COLIV_1-10_dapi.tif]

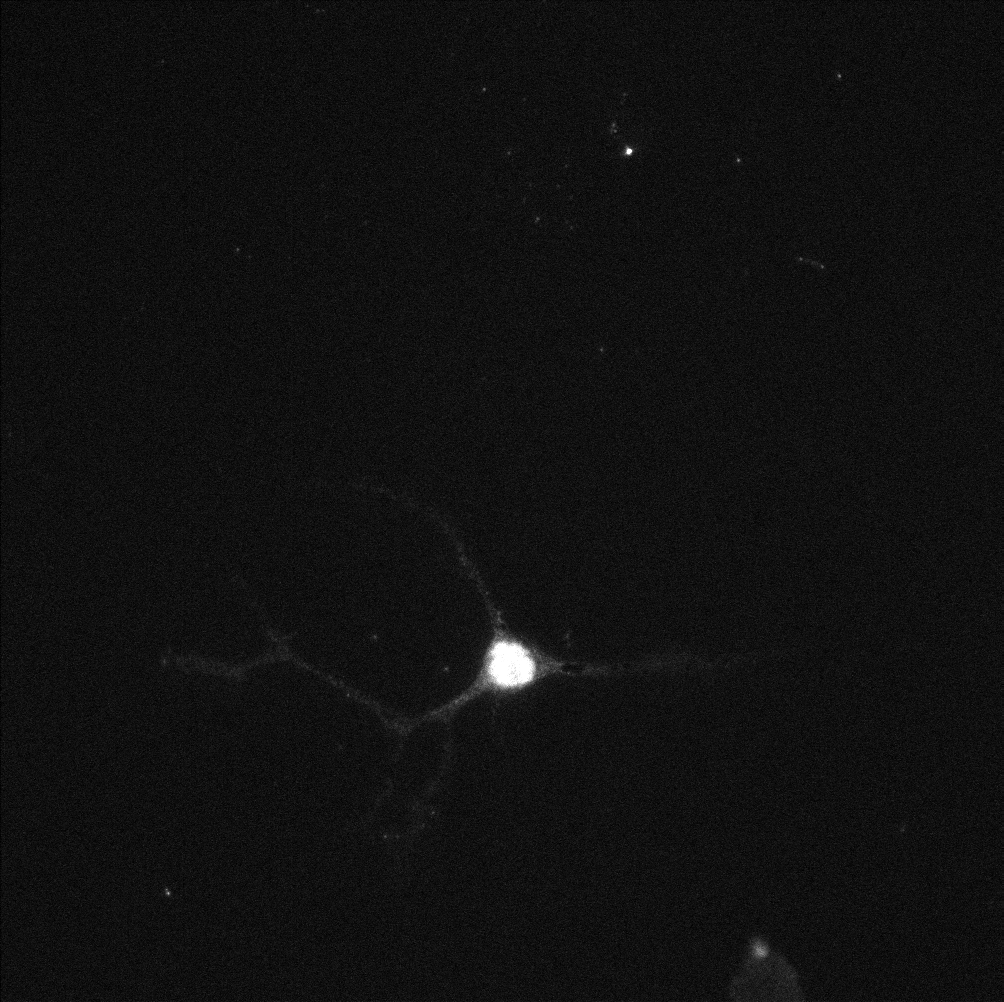

Supplement: Supplementary file 5 — Source data Fig. 4 [file 44319_2025_583_MOESM5_ESM.zip › Figure 4/4A/4A_COLIV_1-10_MITF.tif]

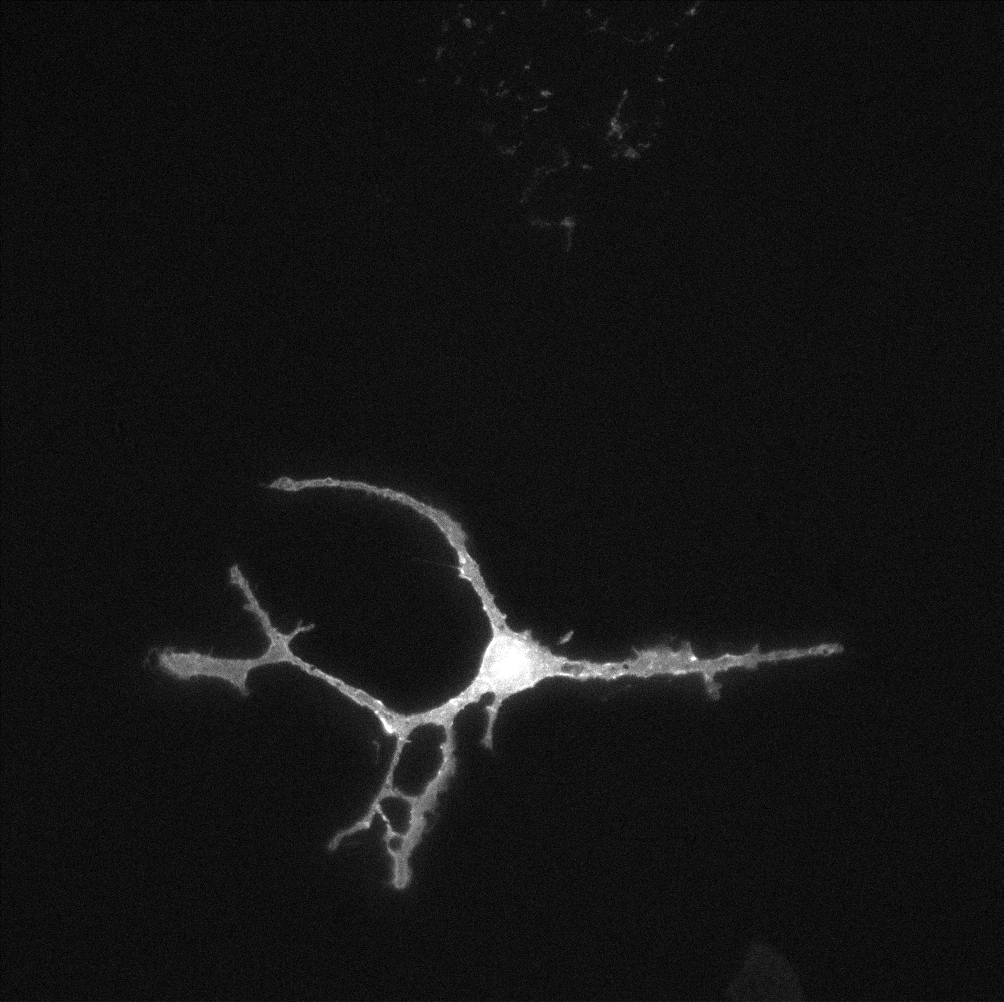

Supplement: Supplementary file 5 — Source data Fig. 4 [file 44319_2025_583_MOESM5_ESM.zip › Figure 4/4A/4A_COLIV_1-10_td-Tomato.tif]

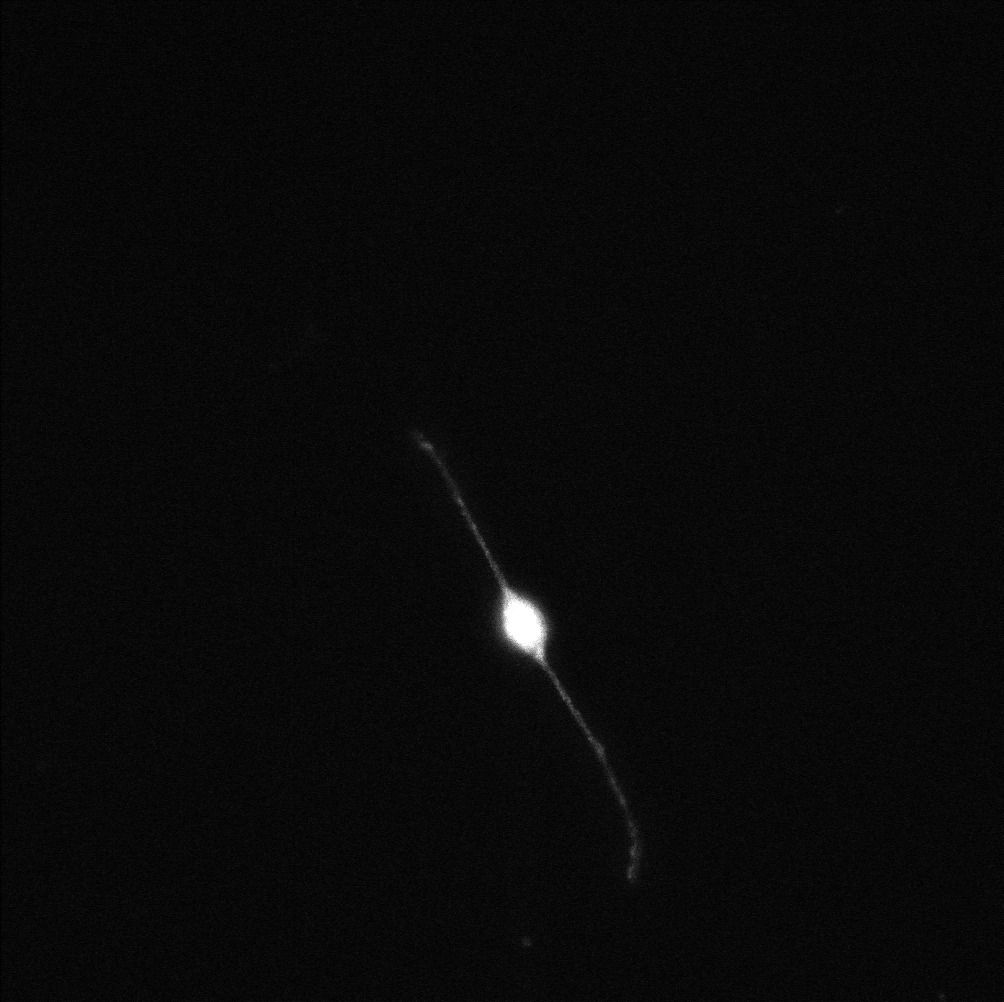

Supplement: Supplementary file 5 — Source data Fig. 4 [file 44319_2025_583_MOESM5_ESM.zip › Figure 4/4E/4E_COLI_1-10_YAP.tif]

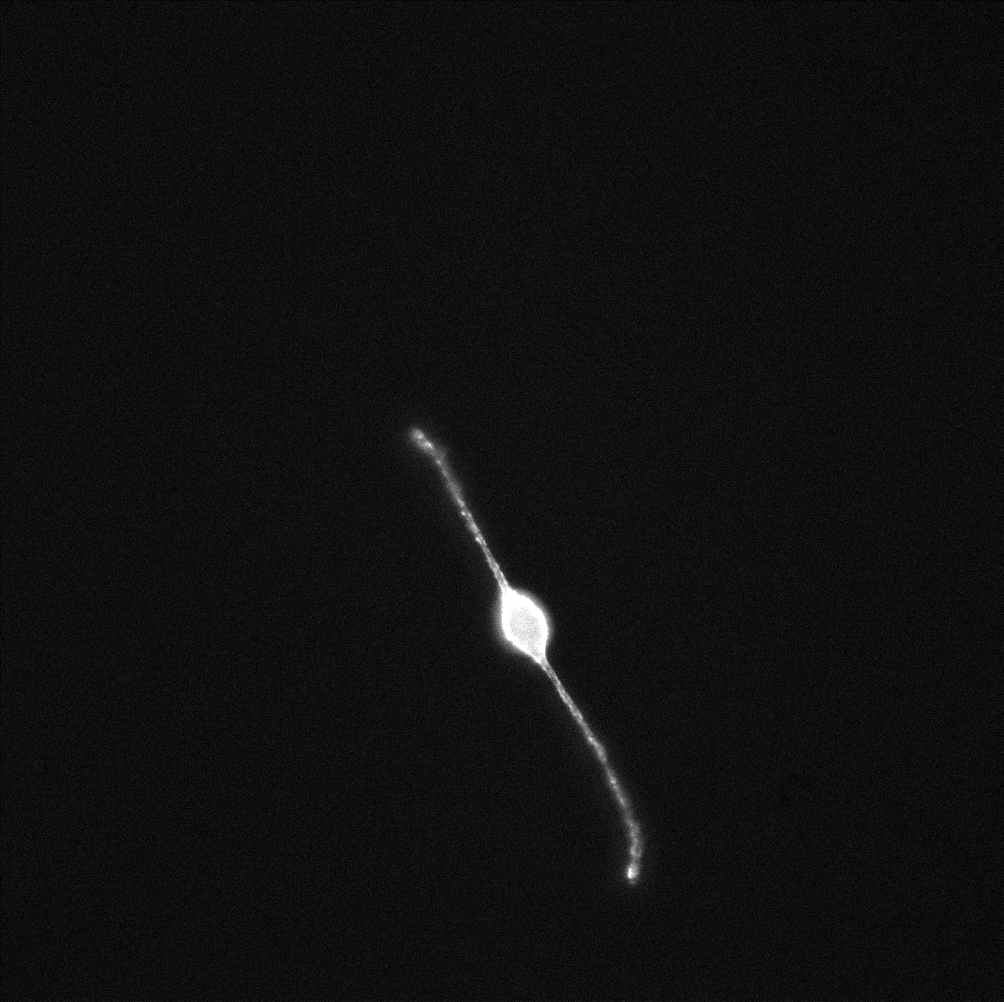

Supplement: Supplementary file 5 — Source data Fig. 4 [file 44319_2025_583_MOESM5_ESM.zip › Figure 4/4E/4E_COLI_1-10_td-Tomato.tif]

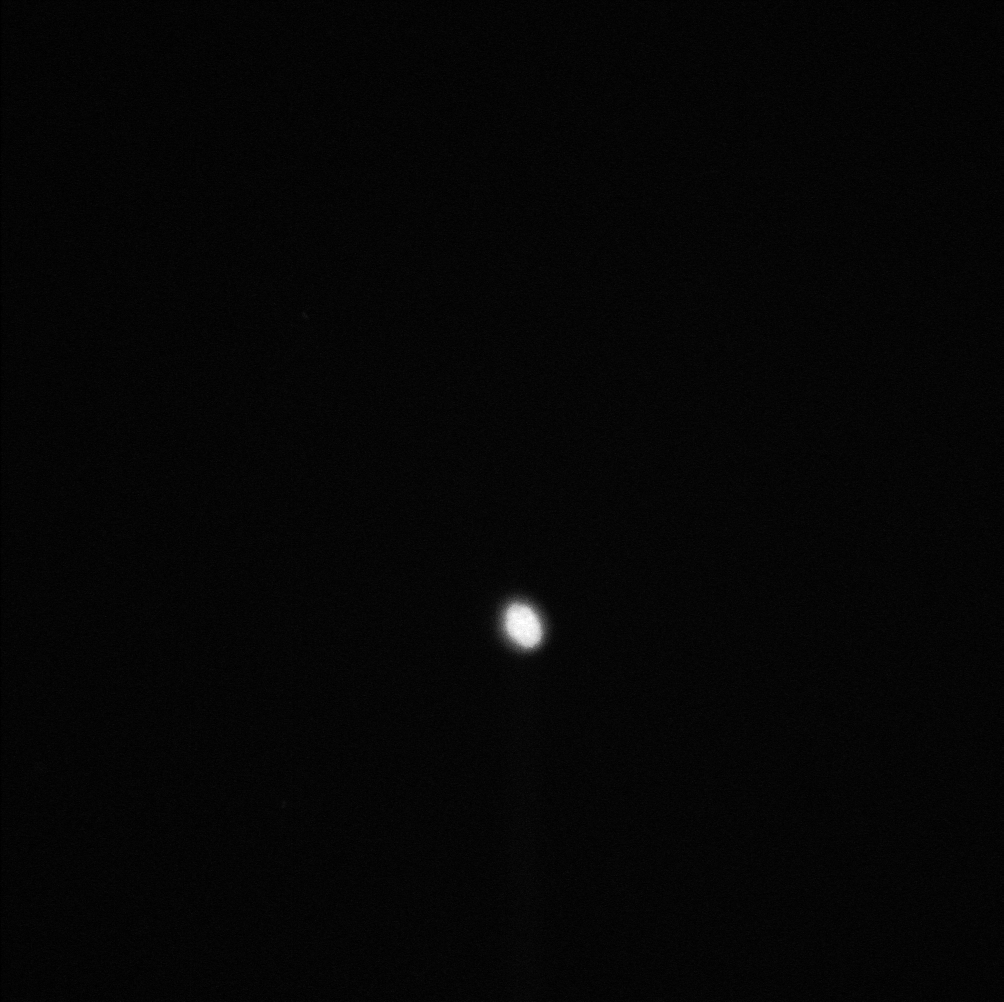

Supplement: Supplementary file 5 — Source data Fig. 4 [file 44319_2025_583_MOESM5_ESM.zip › Figure 4/4E/4E_COLI_1-10_dapi.tif]

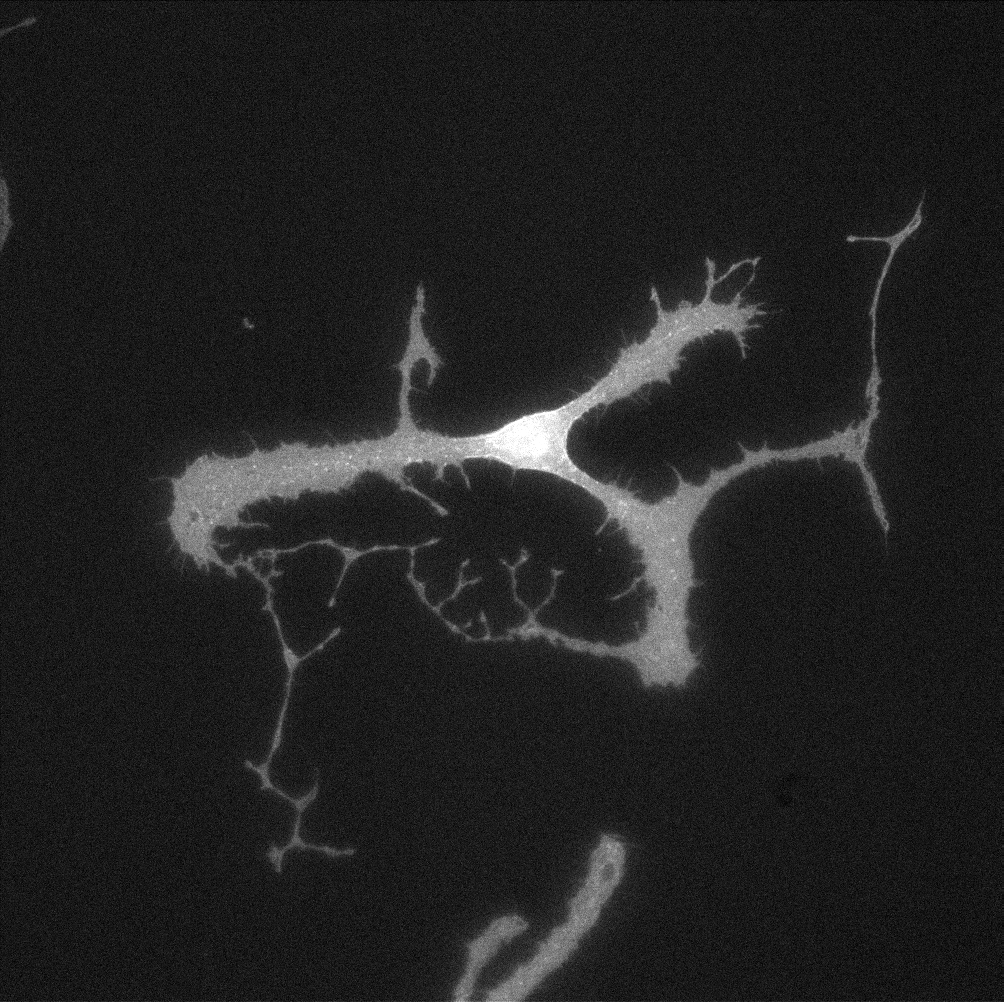

Supplement: Supplementary file 5 — Source data Fig. 4 [file 44319_2025_583_MOESM5_ESM.zip › Figure 4/4E/4E_FN_1-10_td-Tomato.tif]

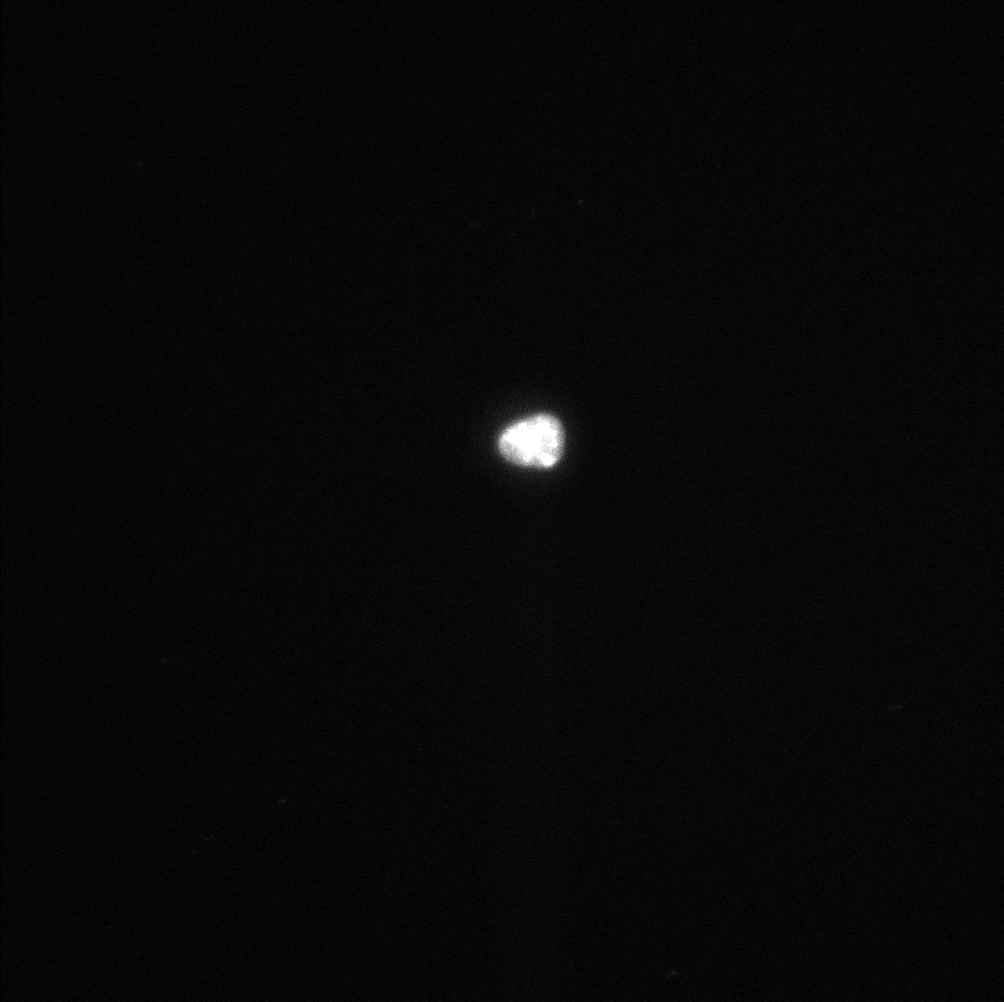

Supplement: Supplementary file 5 — Source data Fig. 4 [file 44319_2025_583_MOESM5_ESM.zip › Figure 4/4E/4E_FN_1-10_dapi.tif]

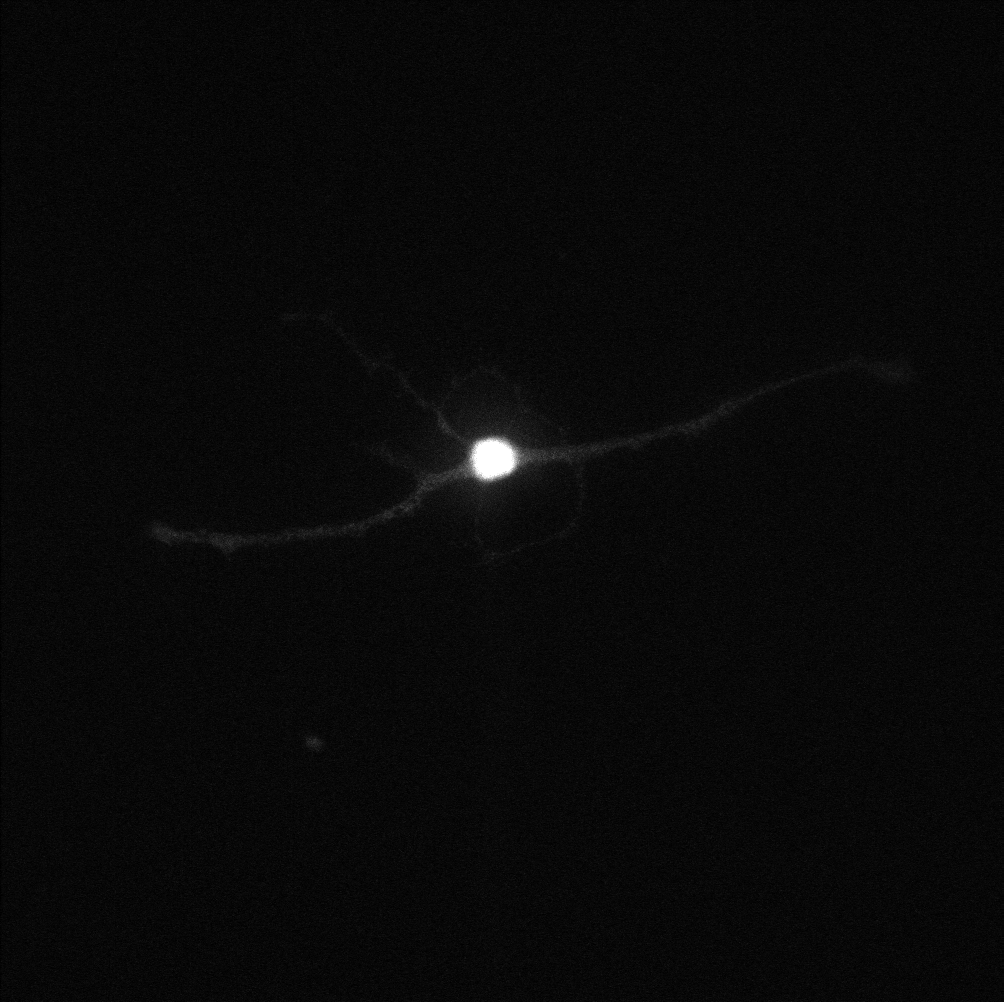

Supplement: Supplementary file 5 — Source data Fig. 4 [file 44319_2025_583_MOESM5_ESM.zip › Figure 4/4E/4E_COLIV_1-10_YAP.tif]

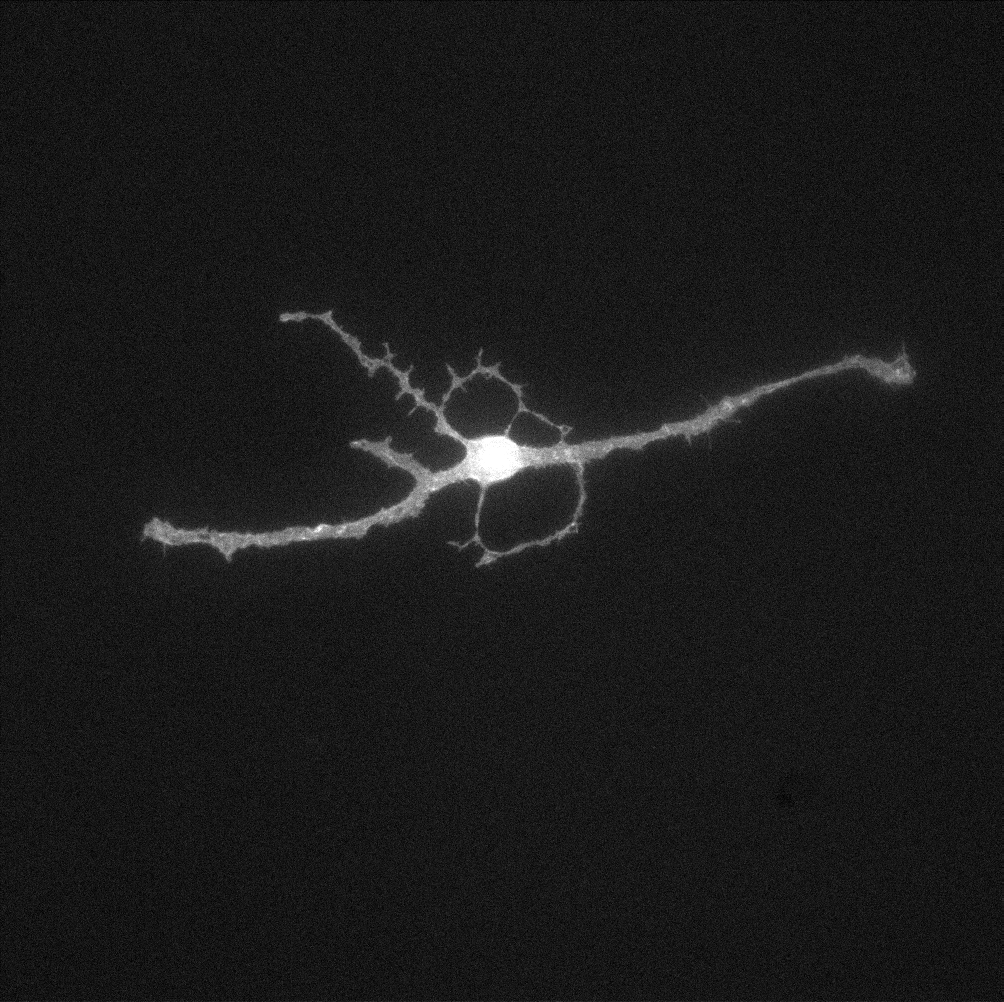

Supplement: Supplementary file 5 — Source data Fig. 4 [file 44319_2025_583_MOESM5_ESM.zip › Figure 4/4E/4E_COLIV_1-10_td-Tomato.tif]

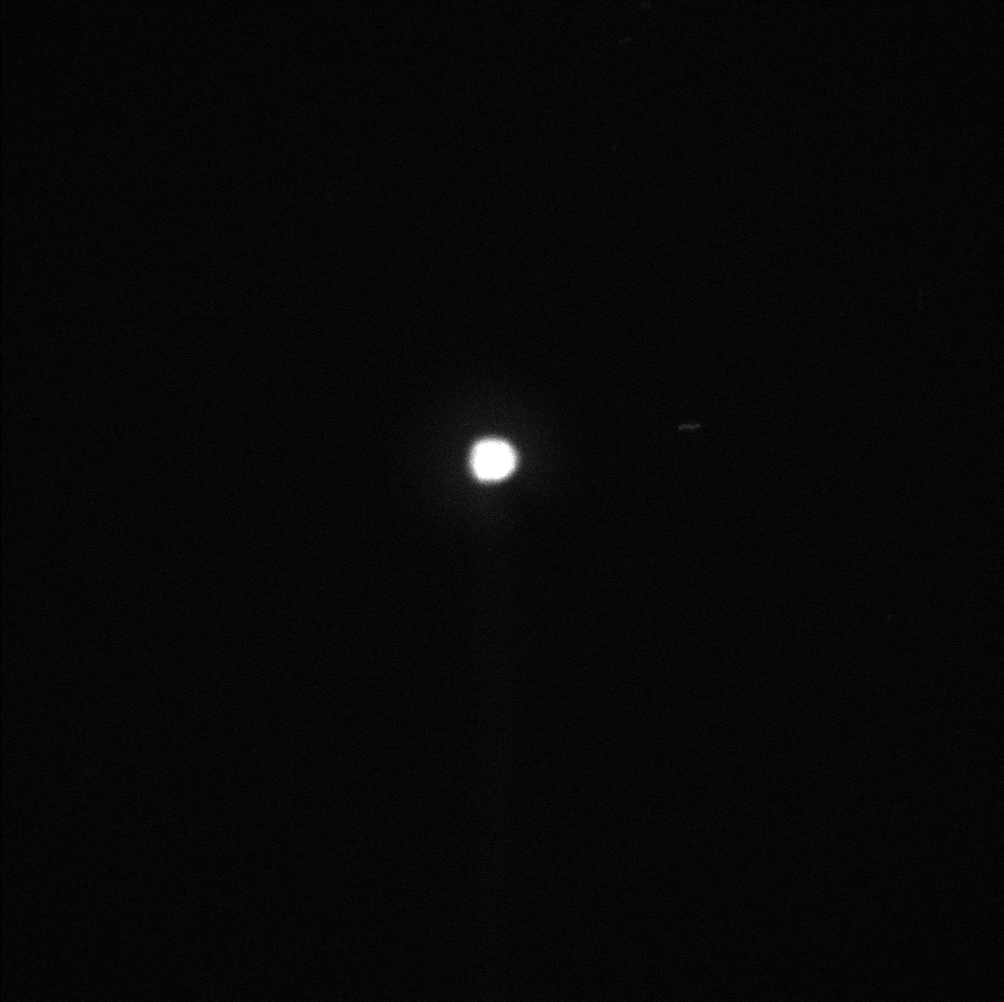

Supplement: Supplementary file 5 — Source data Fig. 4 [file 44319_2025_583_MOESM5_ESM.zip › Figure 4/4E/4E_COLIV_1-10_dapi.tif]

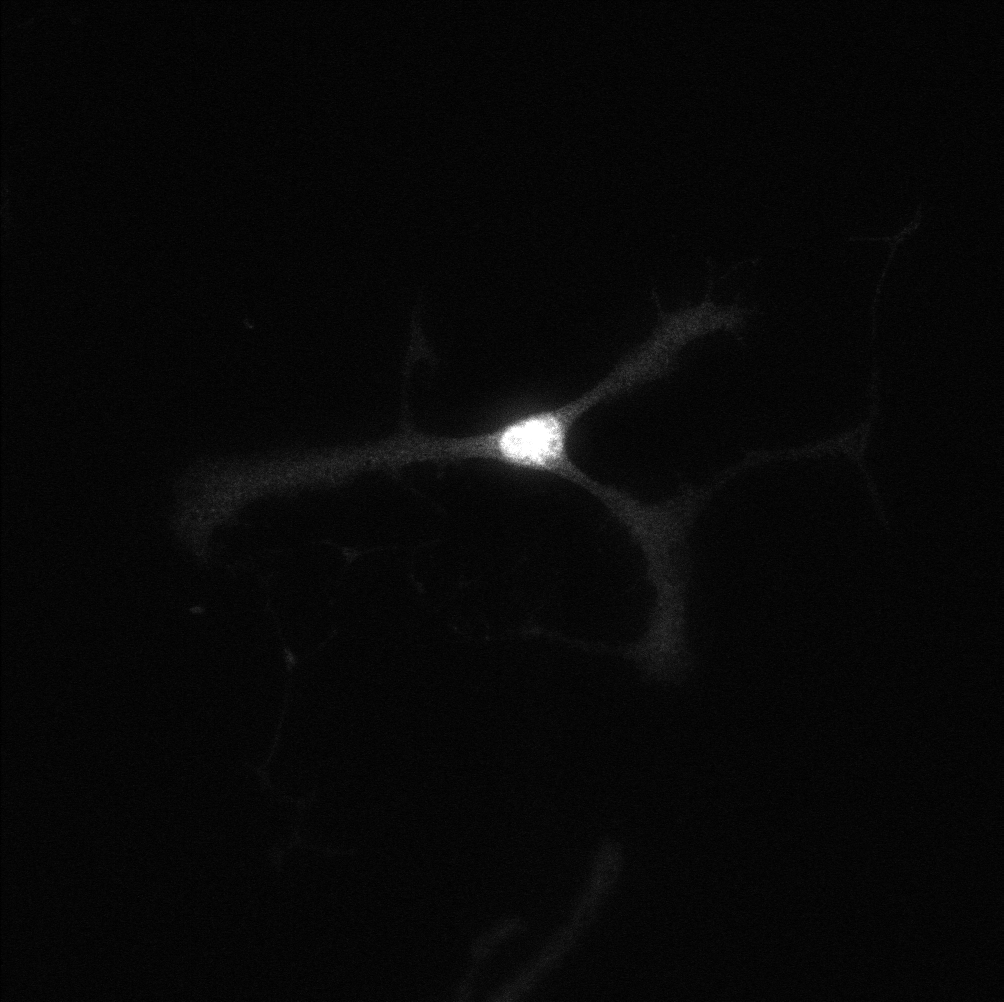

Supplement: Supplementary file 5 — Source data Fig. 4 [file 44319_2025_583_MOESM5_ESM.zip › Figure 4/4E/4E_FN_1-10_YAP.tif]

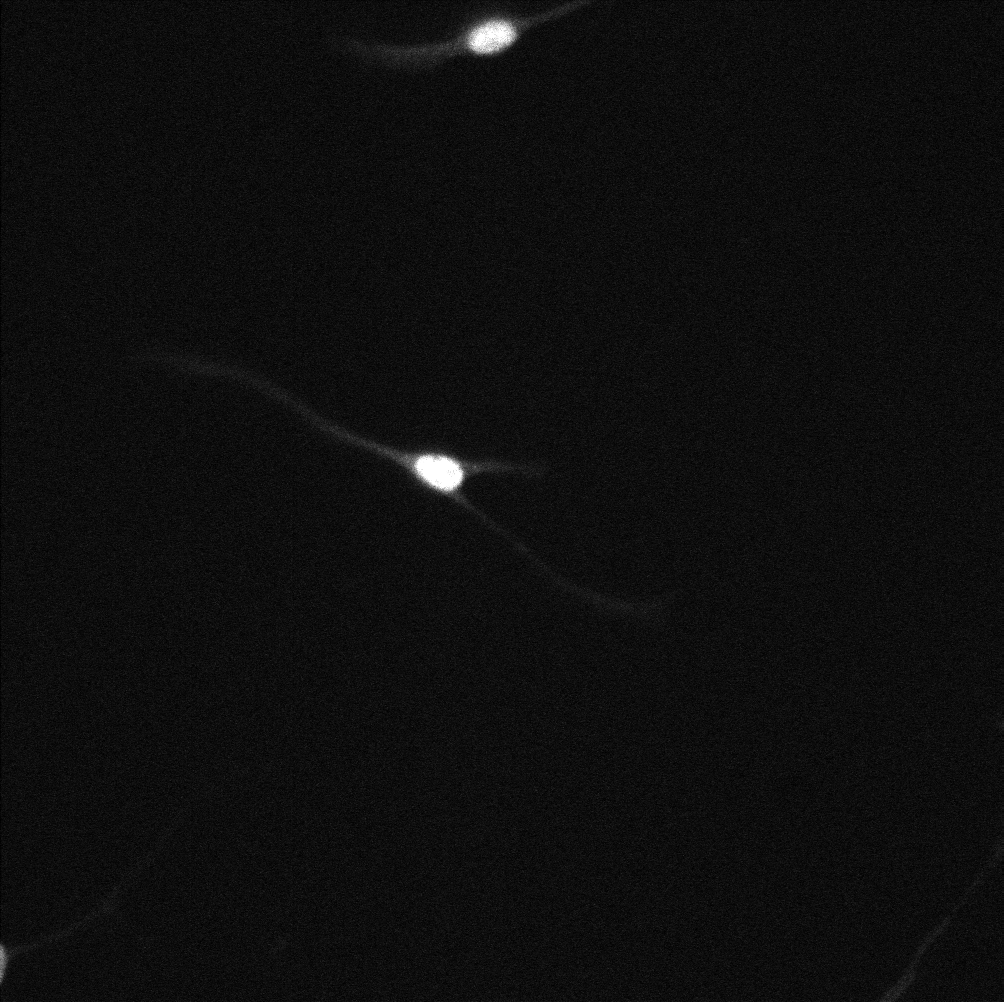

Supplement: Supplementary file 6 — Source data Fig. 5 [file 44319_2025_583_MOESM6_ESM.zip › Figure 6/6G/6G_COLI_1-10_DMSO_MITF.tif]

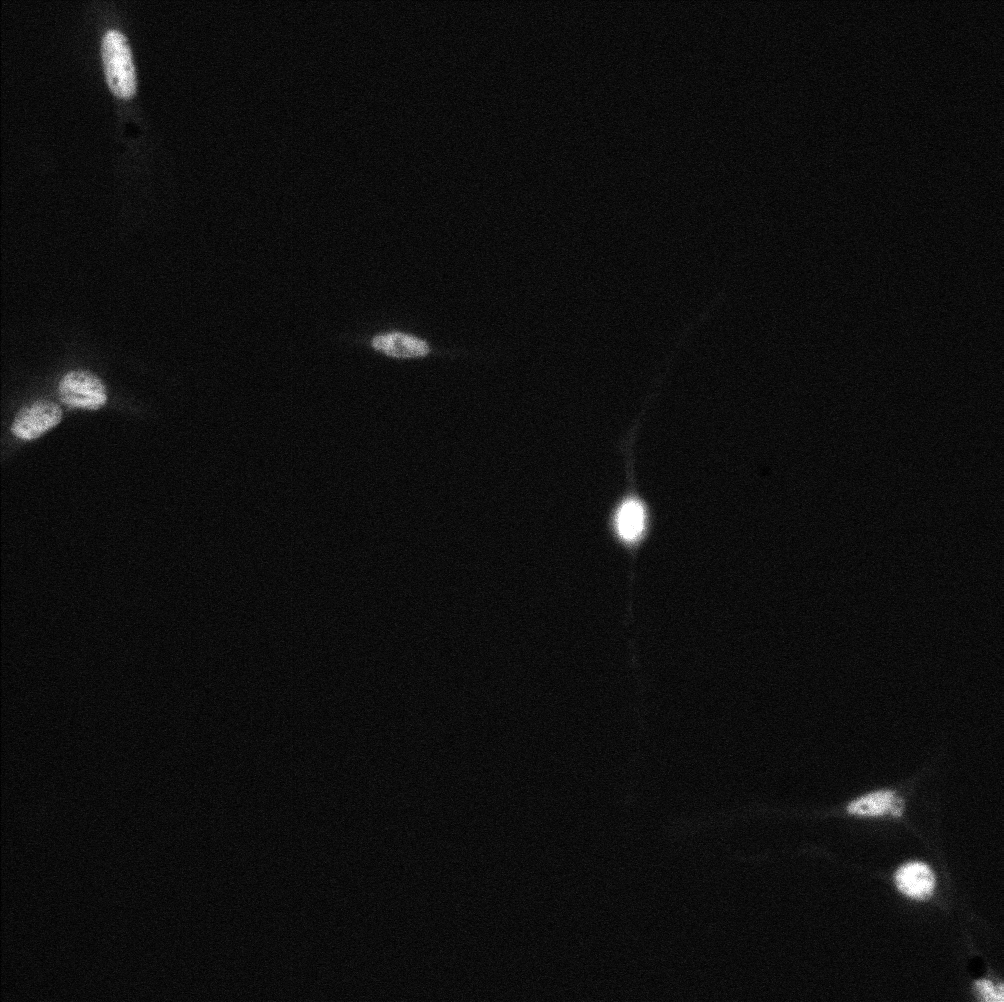

Supplement: Supplementary file 6 — Source data Fig. 5 [file 44319_2025_583_MOESM6_ESM.zip › Figure 6/6G/6G_COLI_1-10_MEKi_MITF.tif]

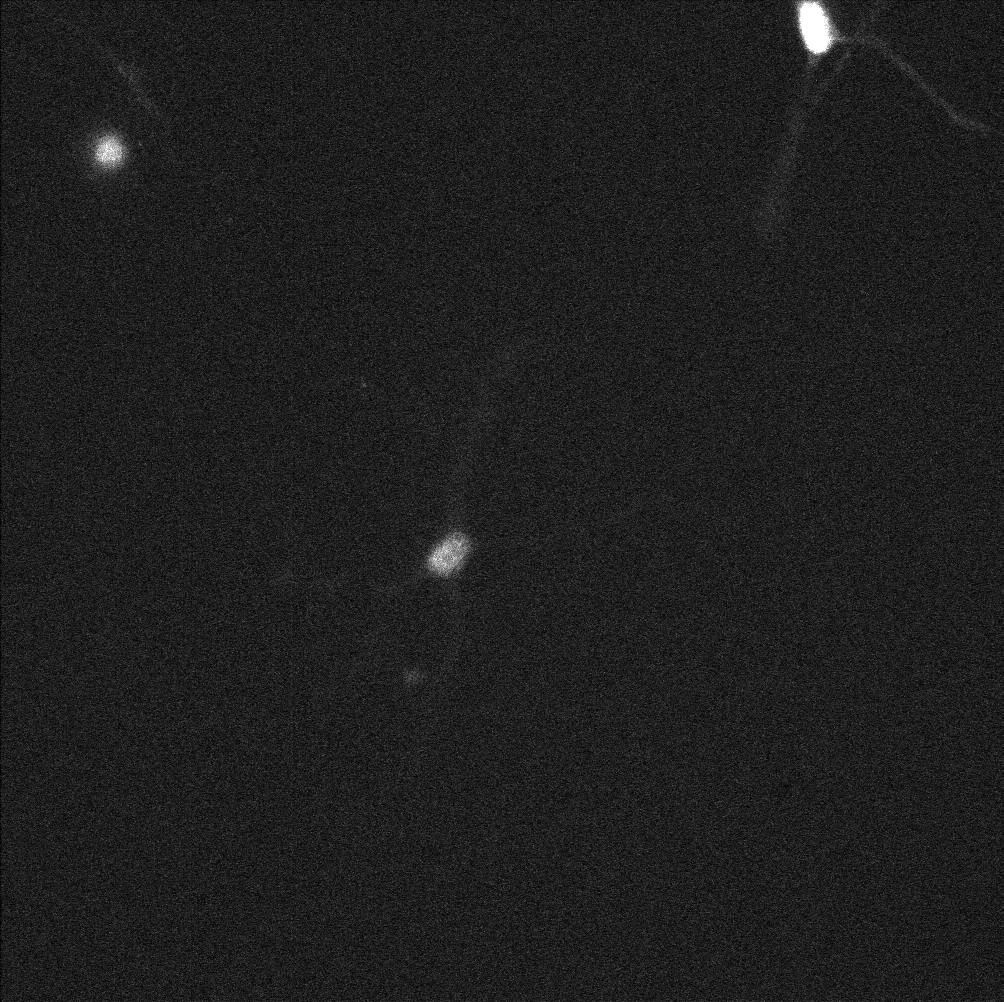

Supplement: Supplementary file 6 — Source data Fig. 5 [file 44319_2025_583_MOESM6_ESM.zip › Figure 6/6G/6G_COLIV_1-10_DMSO_MITF.tif]

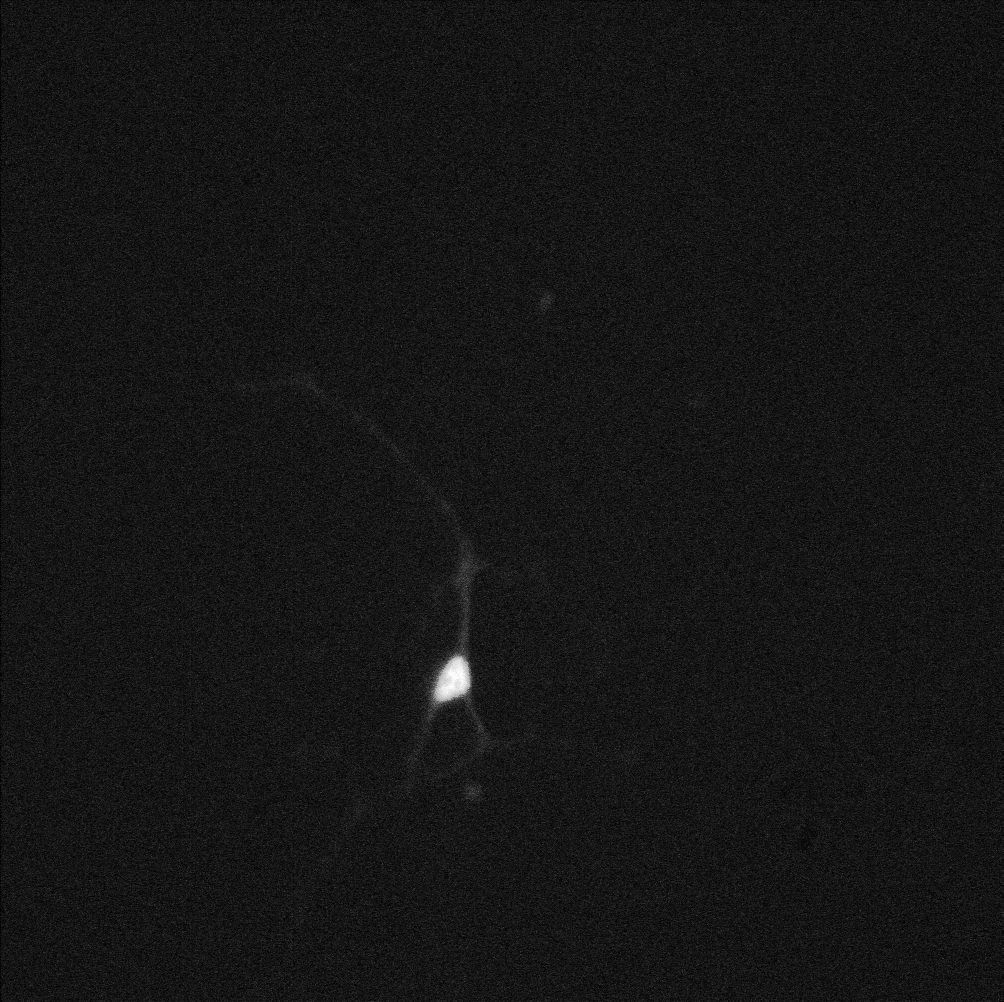

Supplement: Supplementary file 6 — Source data Fig. 5 [file 44319_2025_583_MOESM6_ESM.zip › Figure 6/6G/6G_COLIV_1-10_MEKi_MITF.tif]

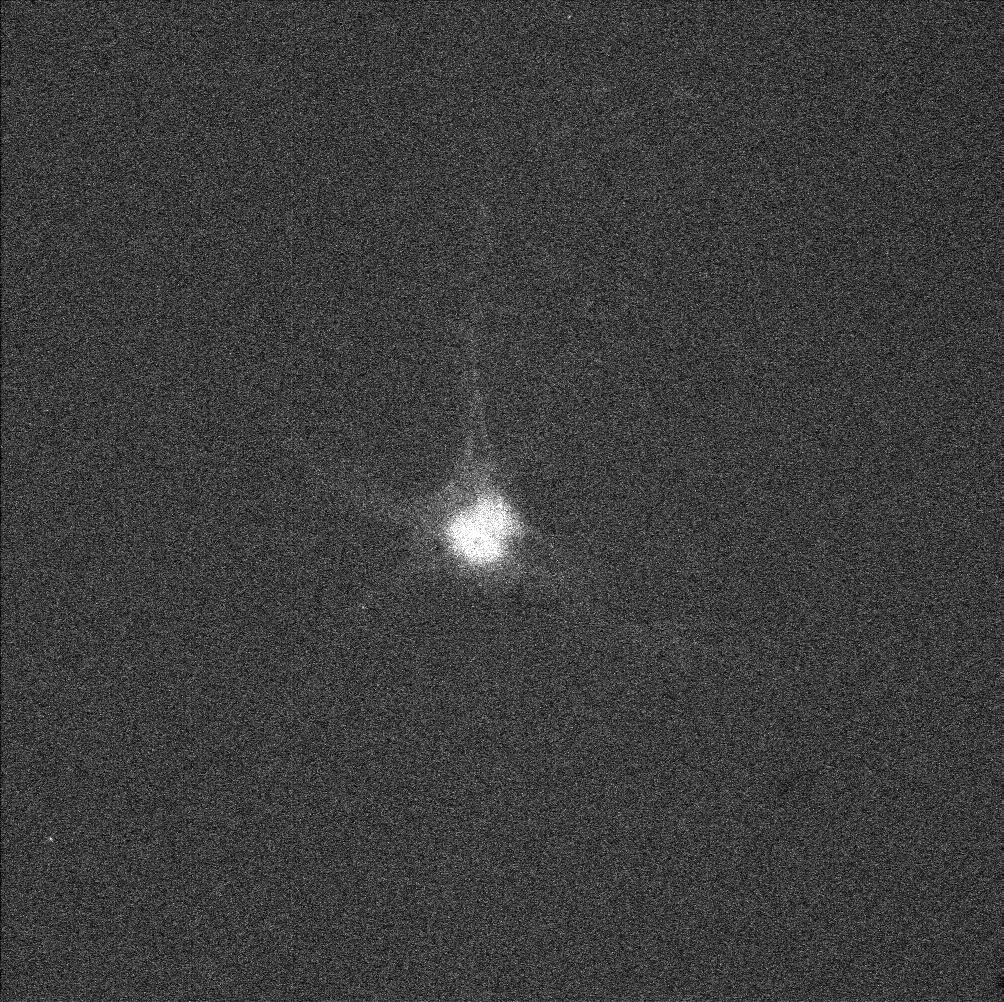

Supplement: Supplementary file 6 — Source data Fig. 5 [file 44319_2025_583_MOESM6_ESM.zip › Figure 6/6G/6G_FN_1-10_DMSO_MITF.tif]

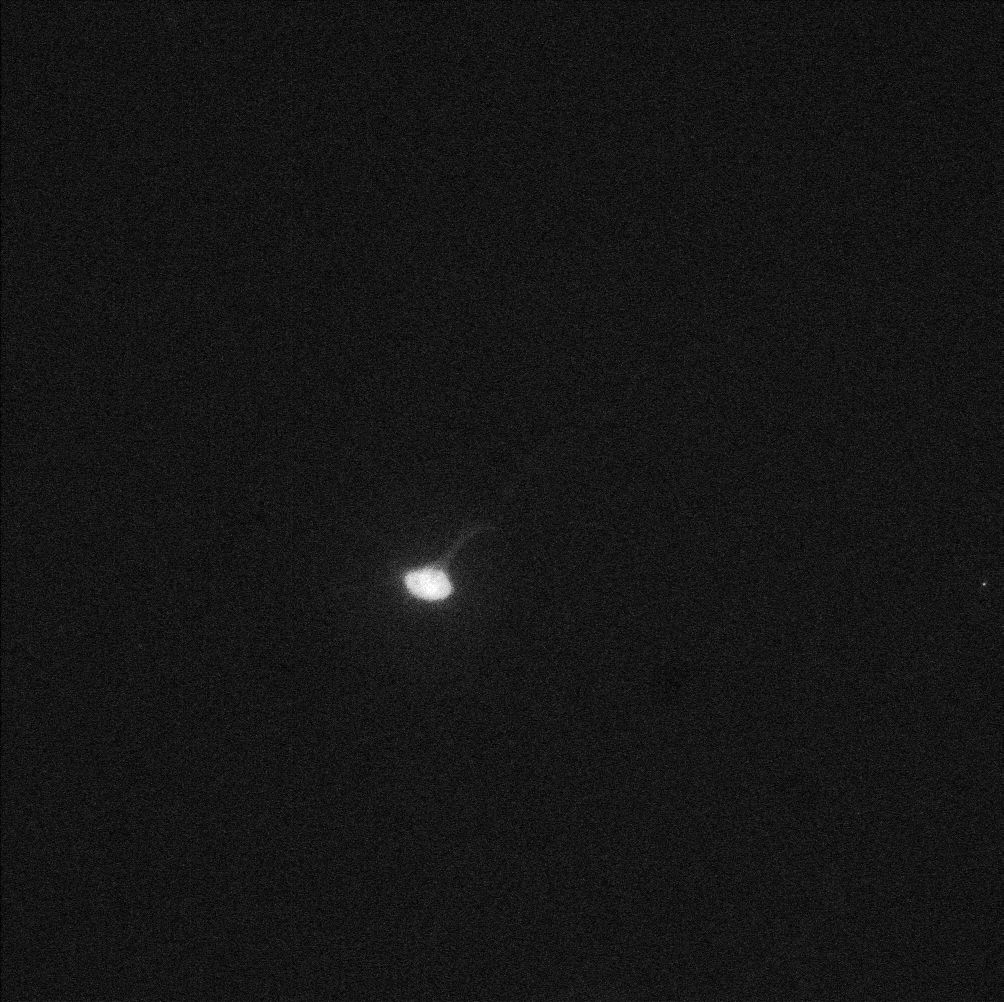

Supplement: Supplementary file 6 — Source data Fig. 5 [file 44319_2025_583_MOESM6_ESM.zip › Figure 6/6G/6G_FN_1-10_MEKi_MITF.tif]

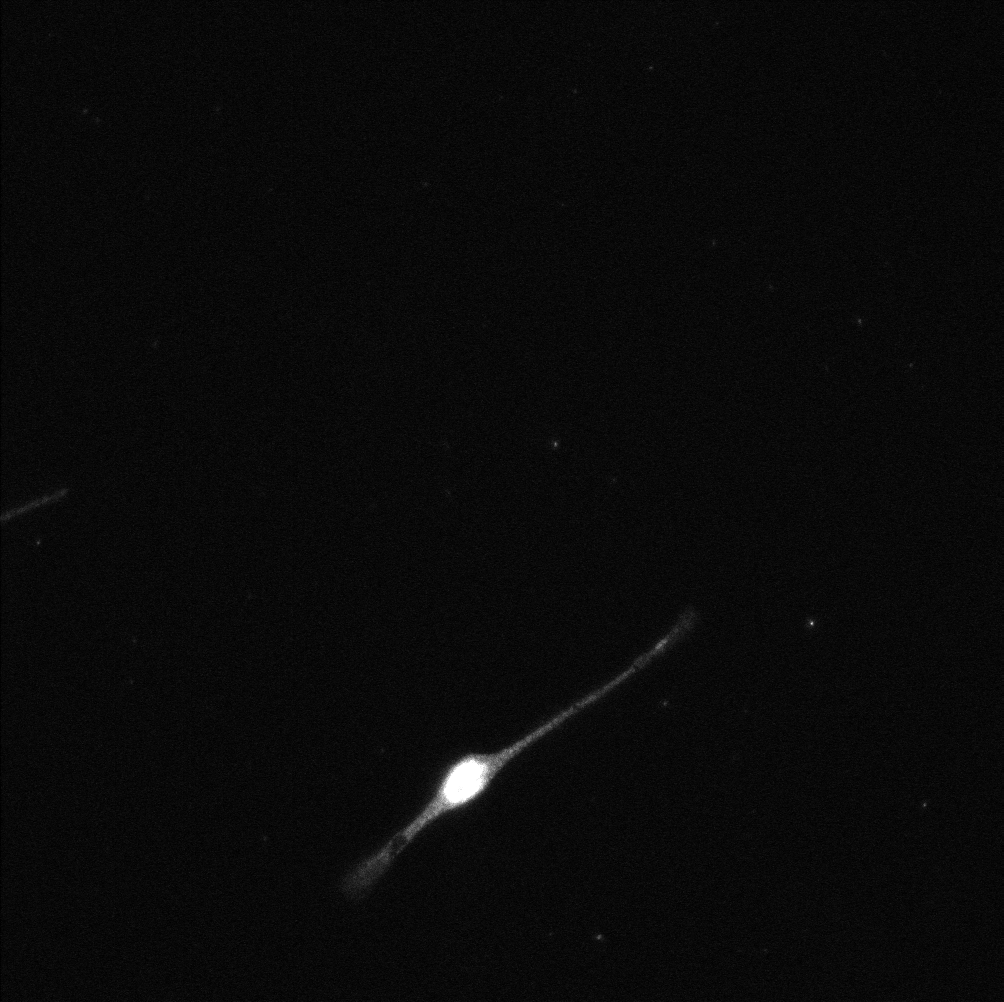

Supplement: Supplementary file 6 — Source data Fig. 5 [file 44319_2025_583_MOESM6_ESM.zip › Figure 6/6J/6J_COLI_1-10_DMSO_MITF.tif]

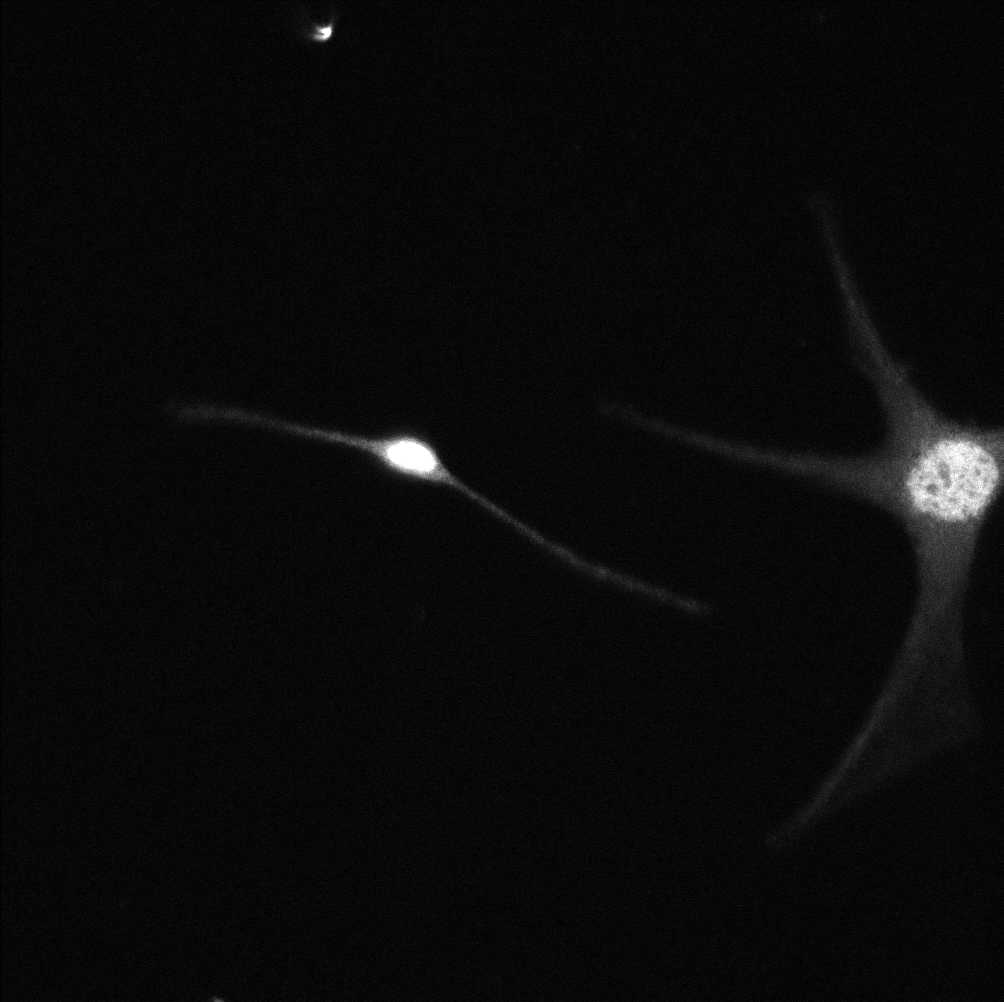

Supplement: Supplementary file 6 — Source data Fig. 5 [file 44319_2025_583_MOESM6_ESM.zip › Figure 6/6J/6J_COLI_1-10_ERKi_MITF.tif]

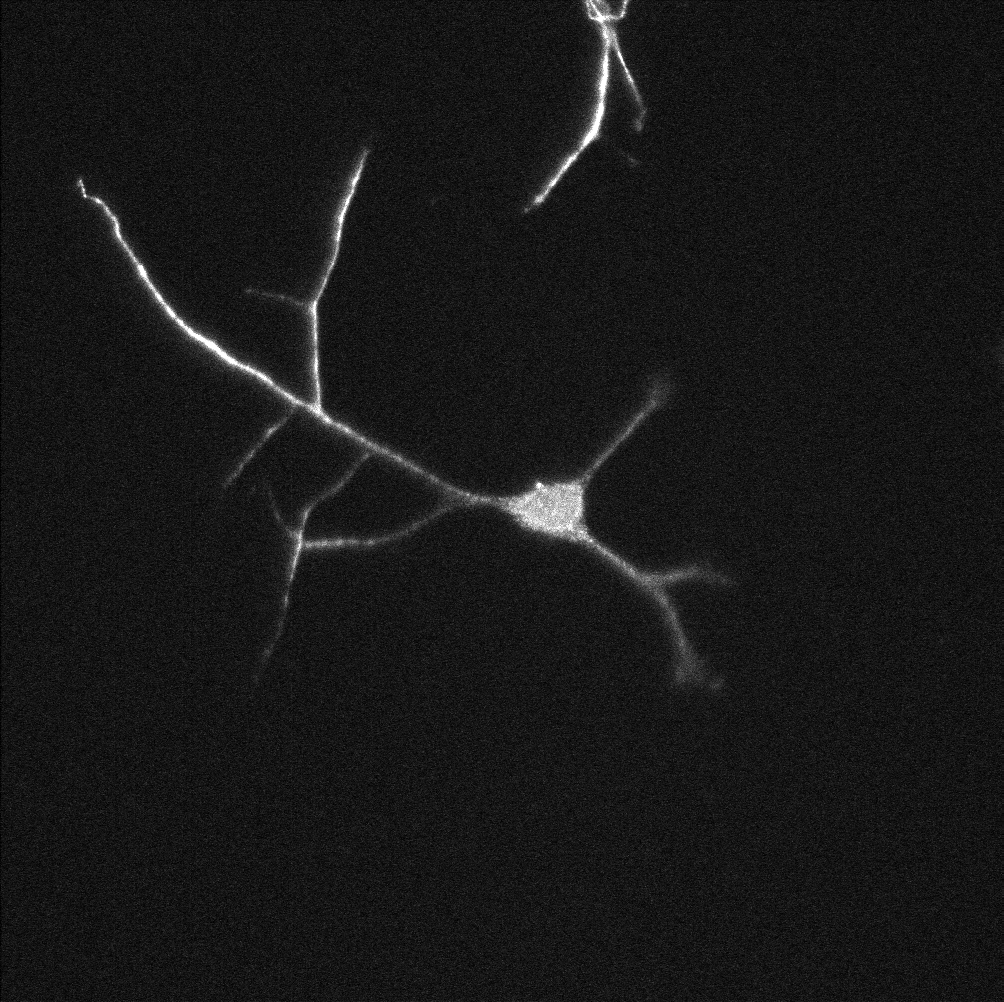

Supplement: Supplementary file 6 — Source data Fig. 5 [file 44319_2025_583_MOESM6_ESM.zip › Figure 6/6J/6J_COLIV_1-10_DMSO_MITF.tif]

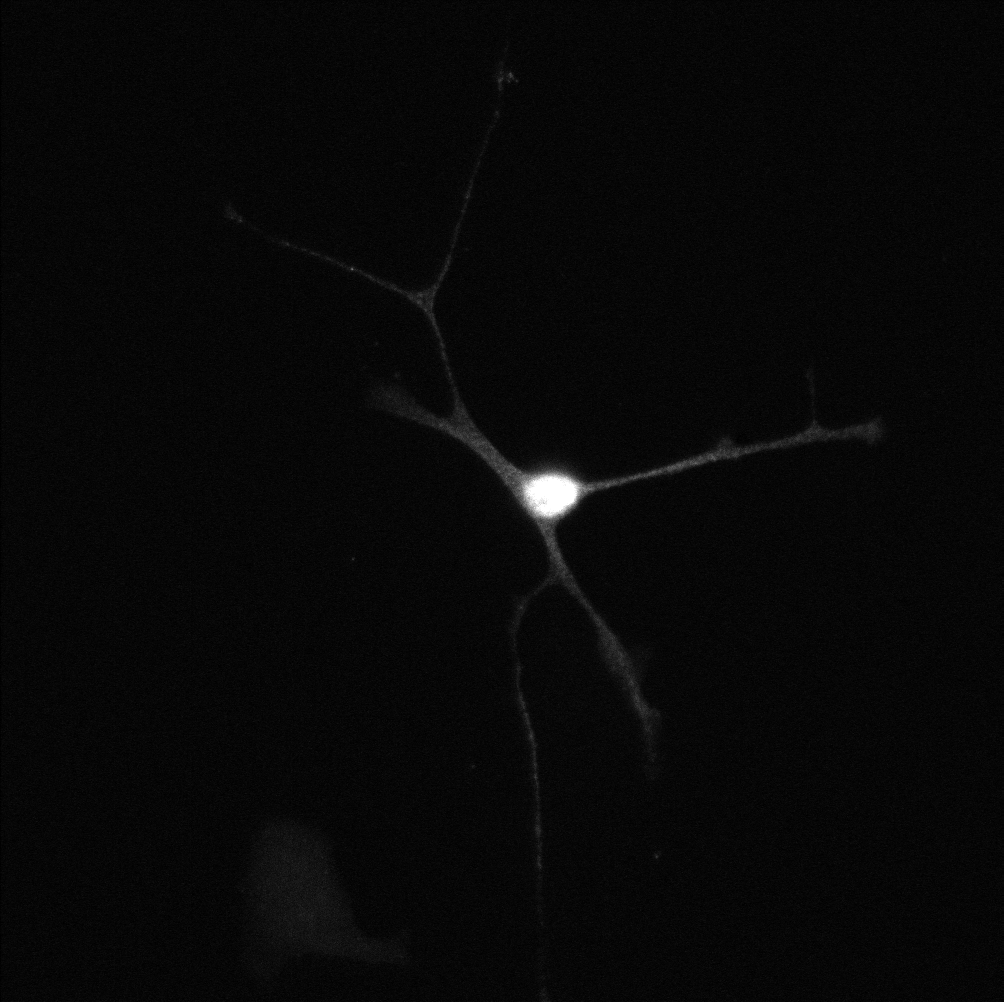

Supplement: Supplementary file 6 — Source data Fig. 5 [file 44319_2025_583_MOESM6_ESM.zip › Figure 6/6J/6J_COLIV_1-10_ERKi_MITF.tif]

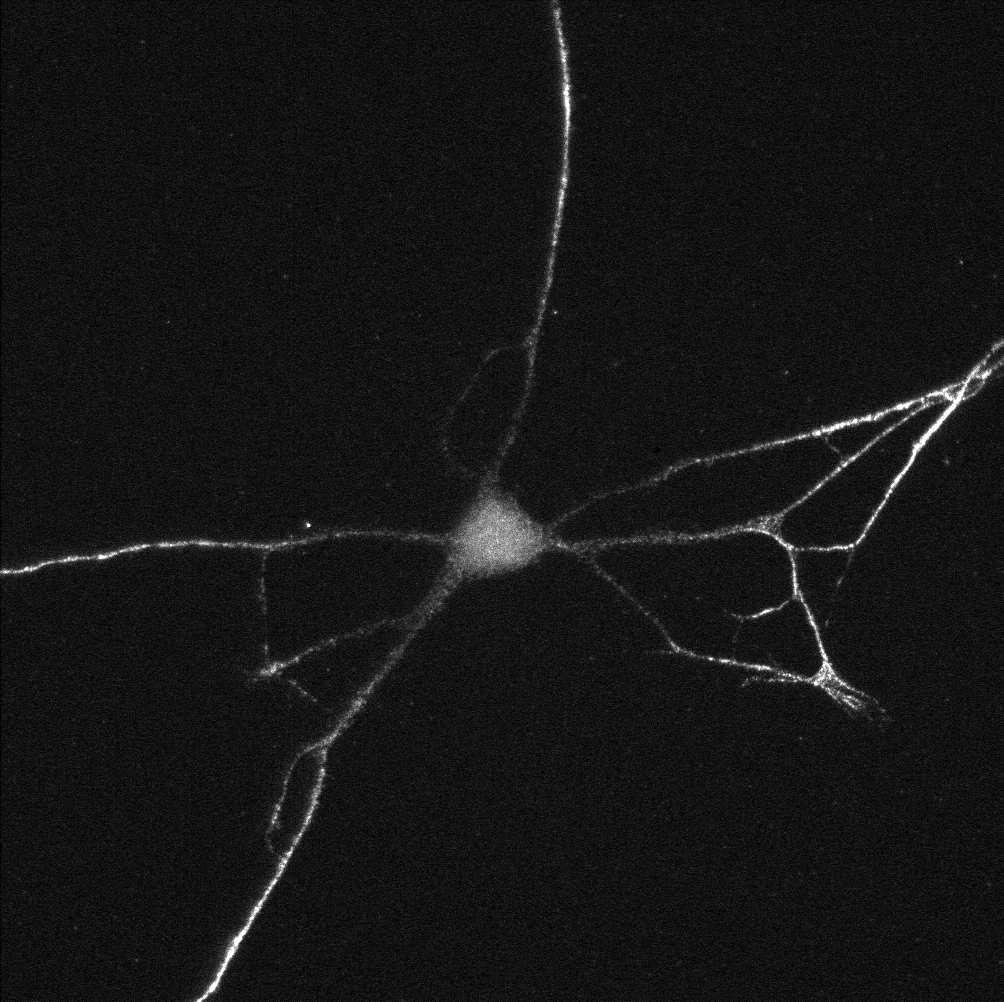

Supplement: Supplementary file 6 — Source data Fig. 5 [file 44319_2025_583_MOESM6_ESM.zip › Figure 6/6J/6J_FN_1-10_DMSO_MITF.tif]

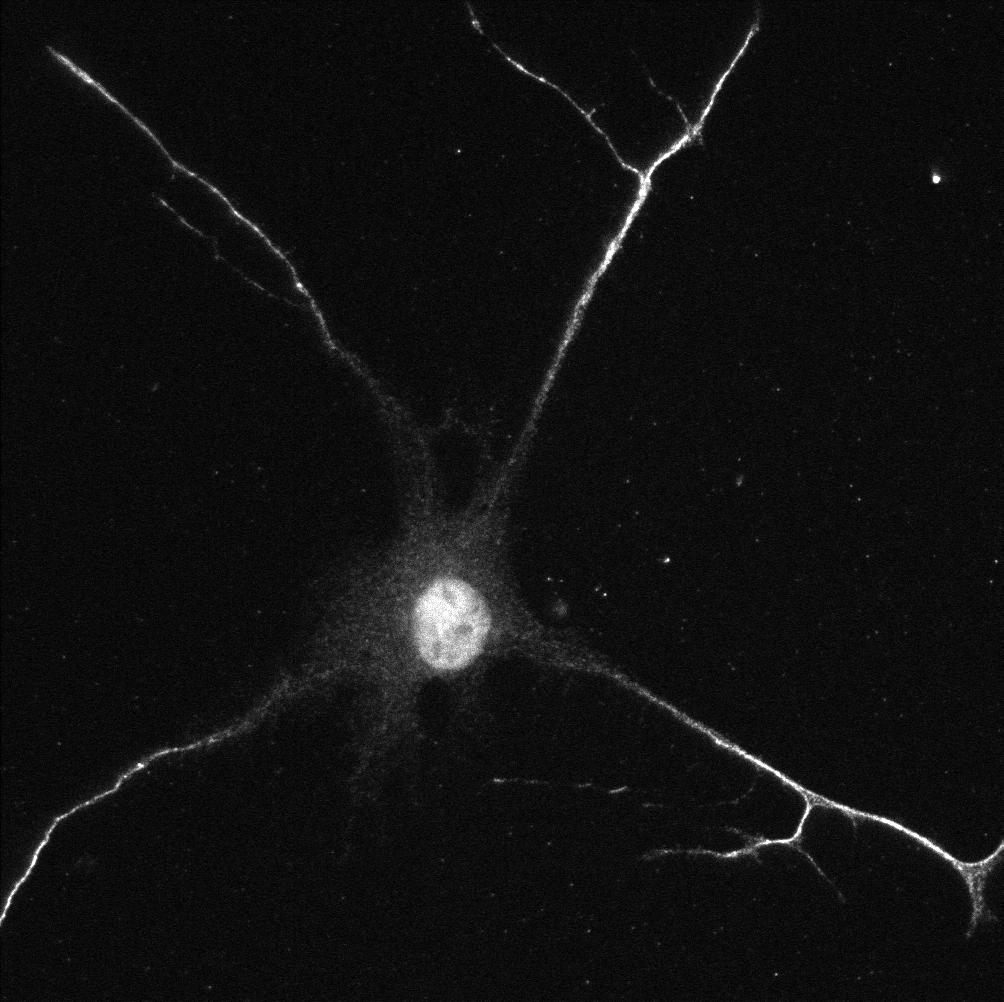

Supplement: Supplementary file 6 — Source data Fig. 5 [file 44319_2025_583_MOESM6_ESM.zip › Figure 6/6J/6J_FN_1-10_ERKi_MITF.tif]

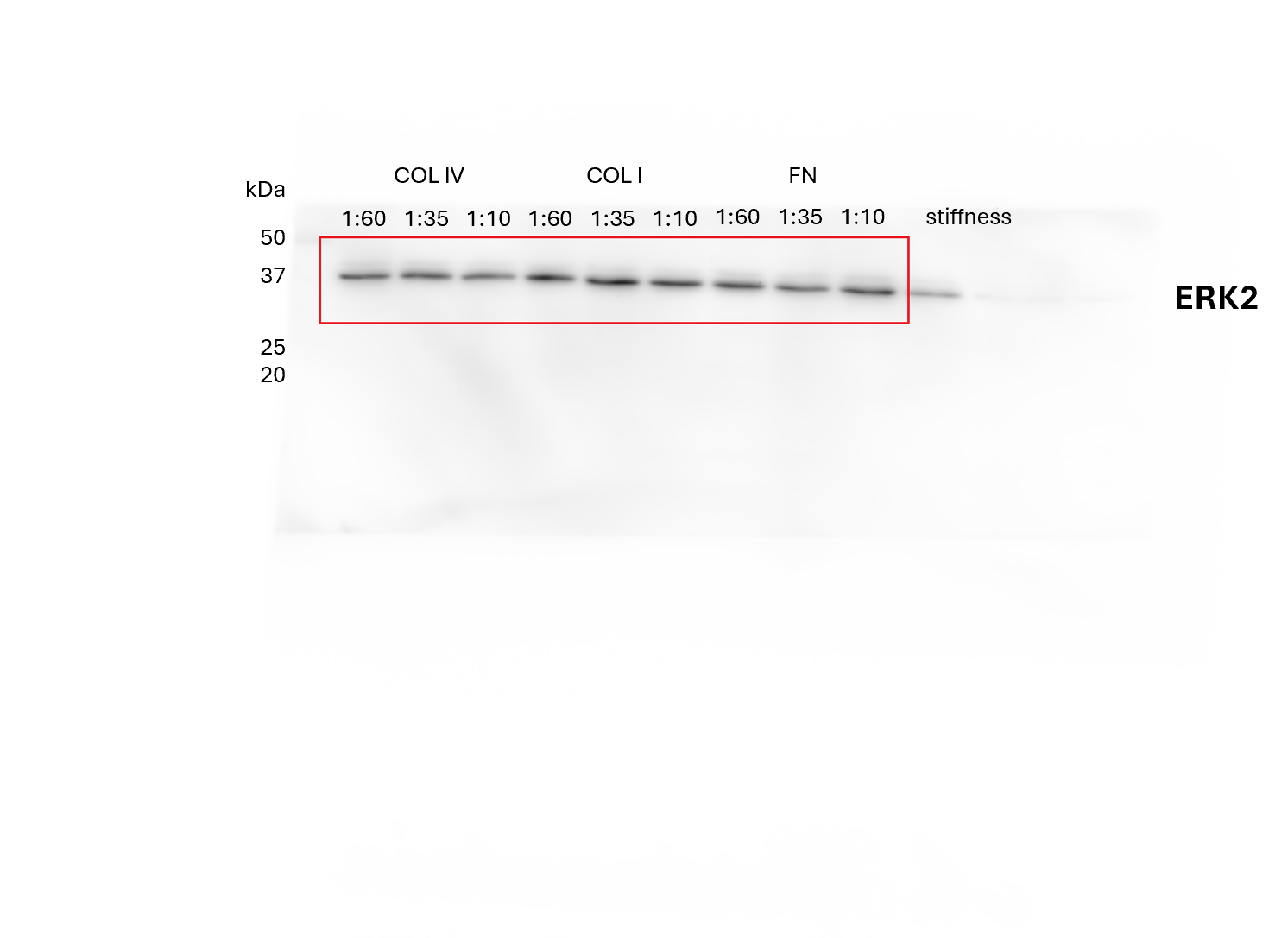

Supplement: Supplementary file 6 — Source data Fig. 5 [file 44319_2025_583_MOESM6_ESM.zip › Figure 6/6E/WB_ERK2.tif]

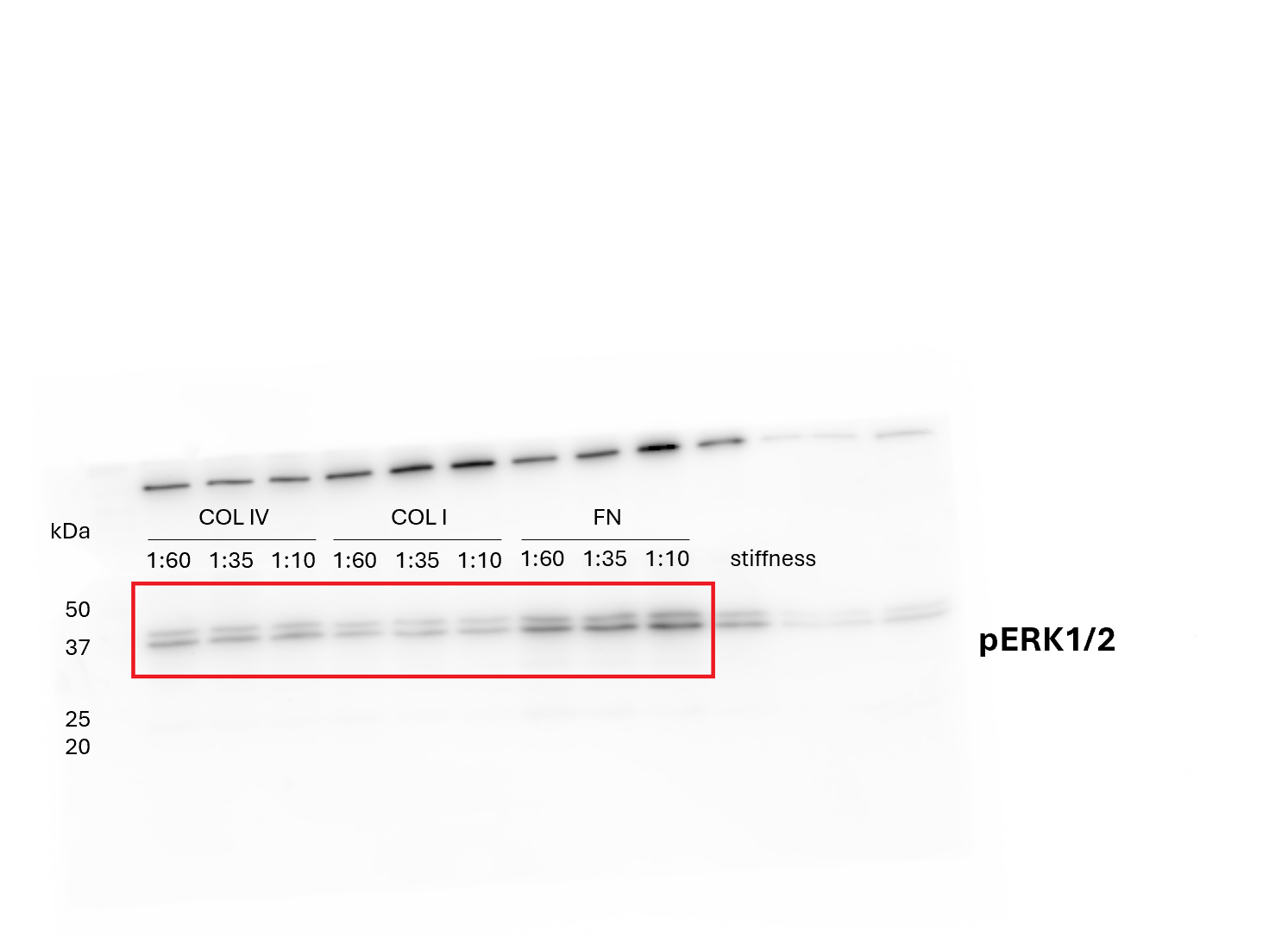

Supplement: Supplementary file 6 — Source data Fig. 5 [file 44319_2025_583_MOESM6_ESM.zip › Figure 6/6E/WB_pERK1-2.tif]

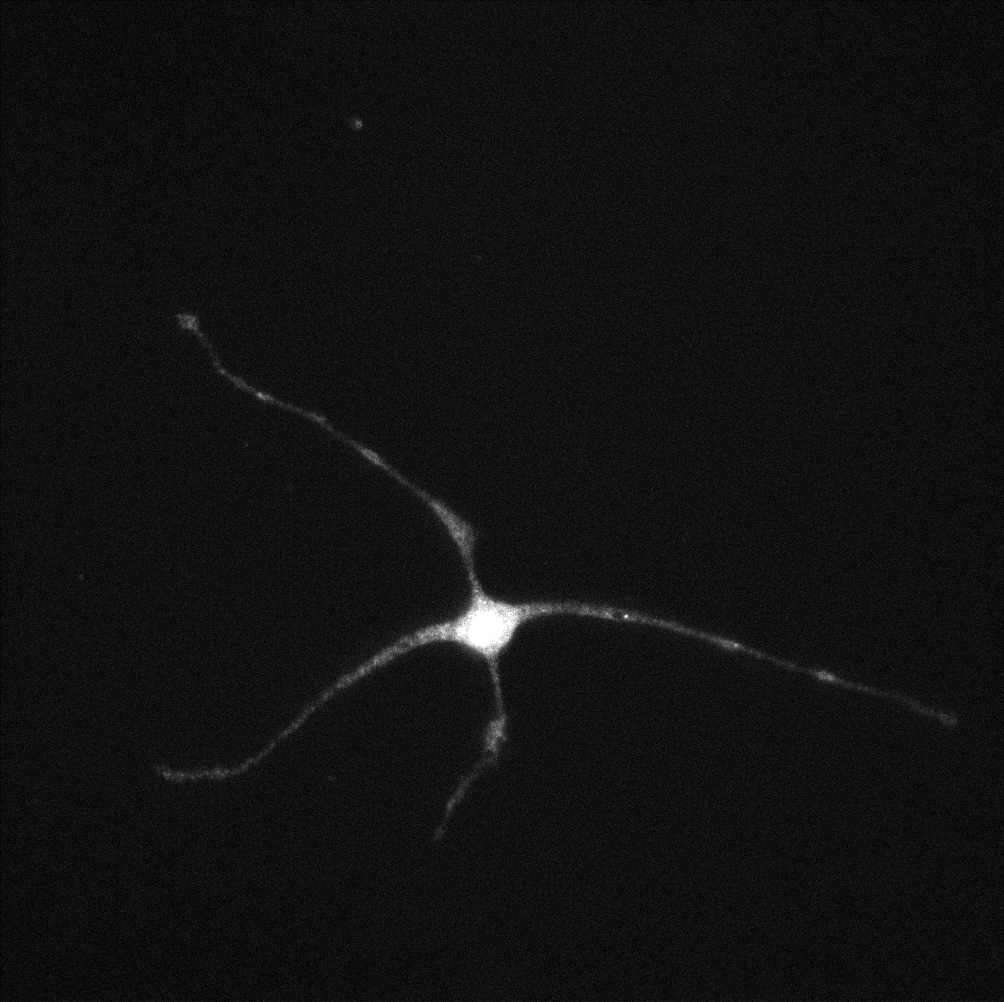

Supplement: Supplementary file 6 — Source data Fig. 5 [file 44319_2025_583_MOESM6_ESM.zip › Figure 6/6M/6M_COLIV_1-10_DMSO_pERK1-2.tif]

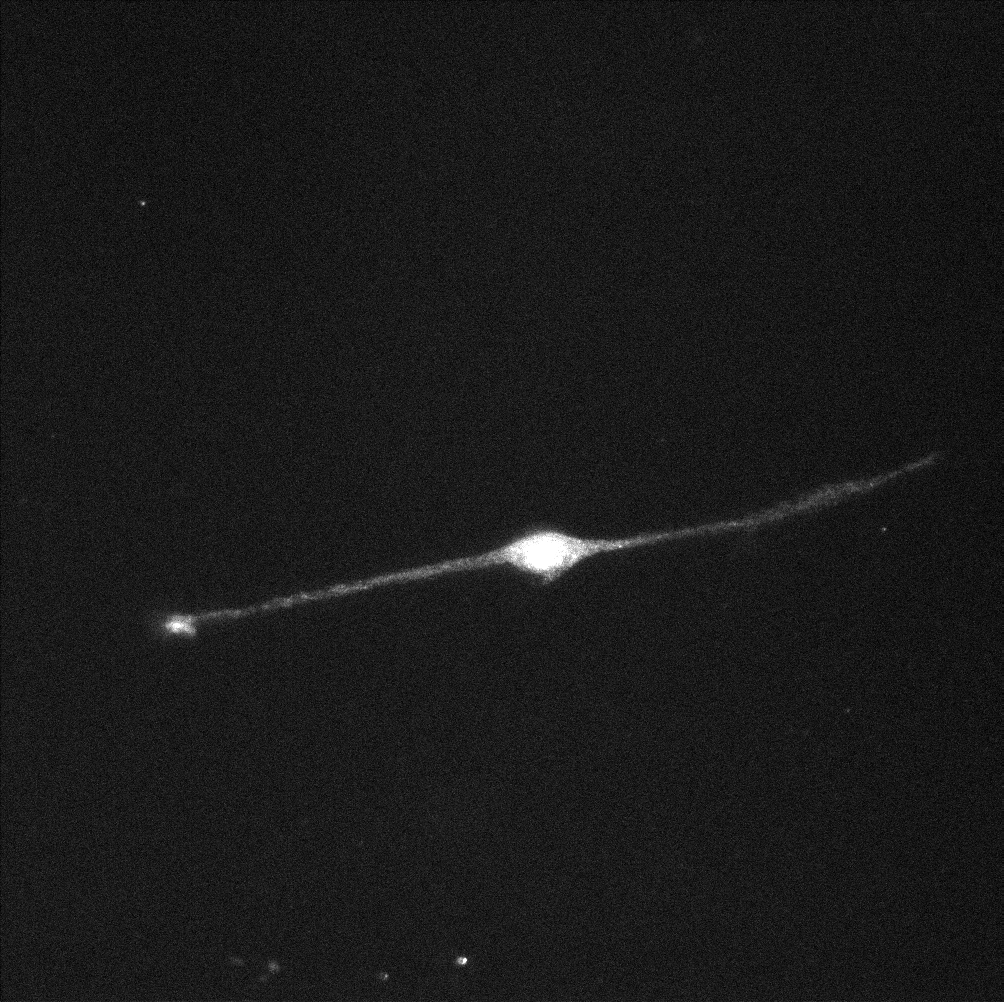

Supplement: Supplementary file 6 — Source data Fig. 5 [file 44319_2025_583_MOESM6_ESM.zip › Figure 6/6M/6M_COLI_1-10_DMSO_pERK1-2.tif]

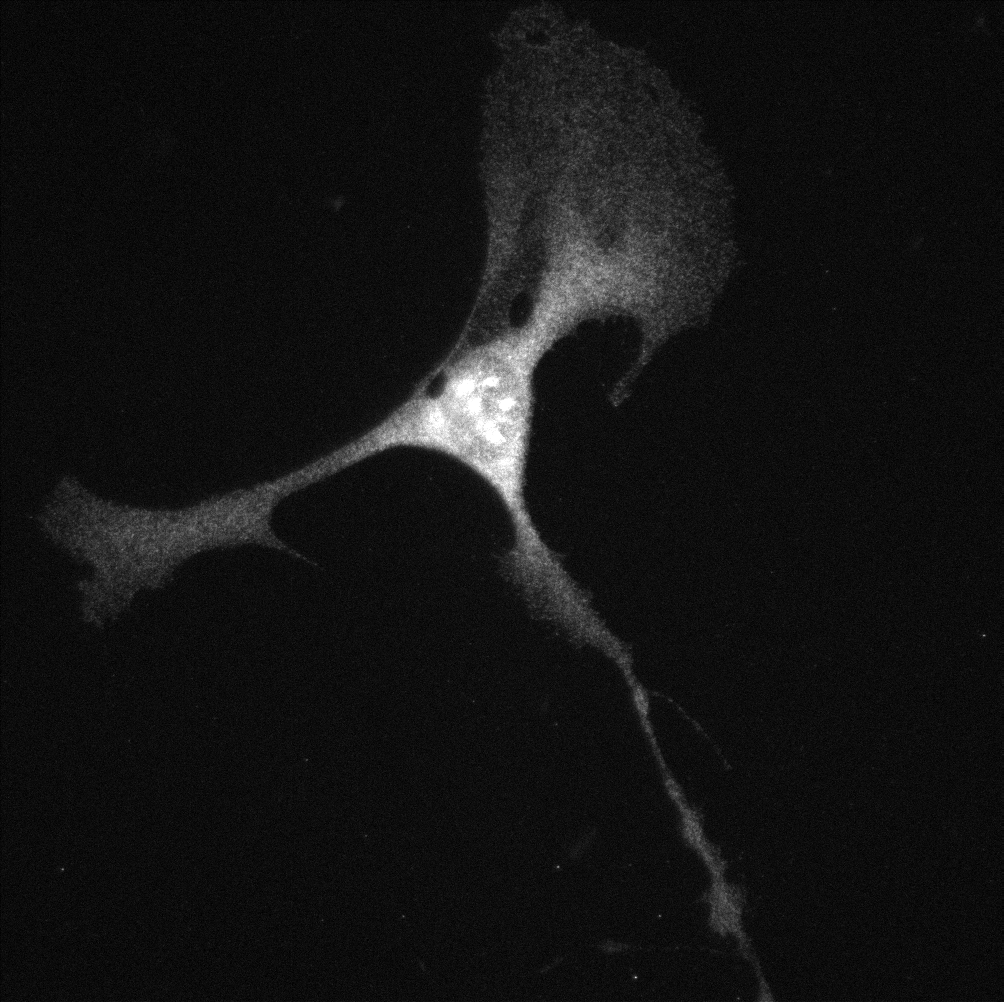

Supplement: Supplementary file 6 — Source data Fig. 5 [file 44319_2025_583_MOESM6_ESM.zip › Figure 6/6M/6M_FN_1-10_DMSO_pERK1-2.tif]

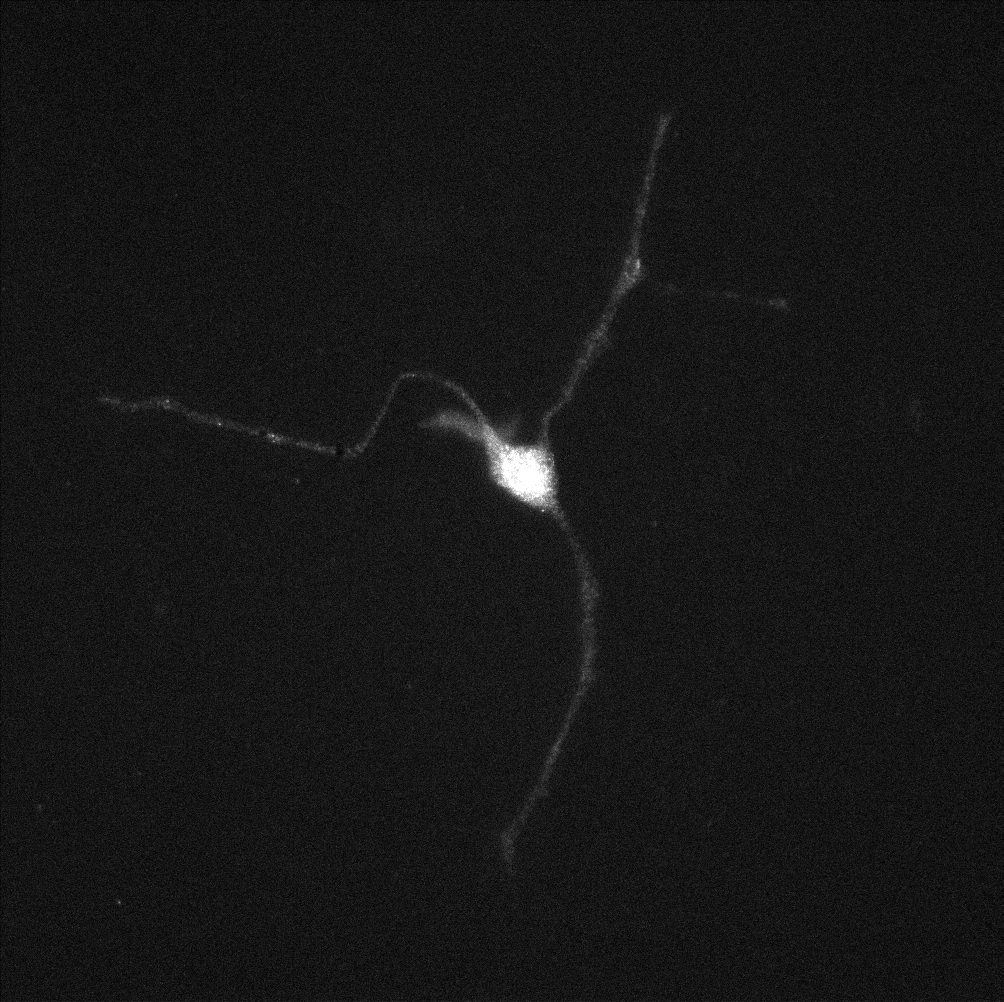

Supplement: Supplementary file 6 — Source data Fig. 5 [file 44319_2025_583_MOESM6_ESM.zip › Figure 6/6M/6M_COLIV_1-10_FAKi_pERK1-2.tif]

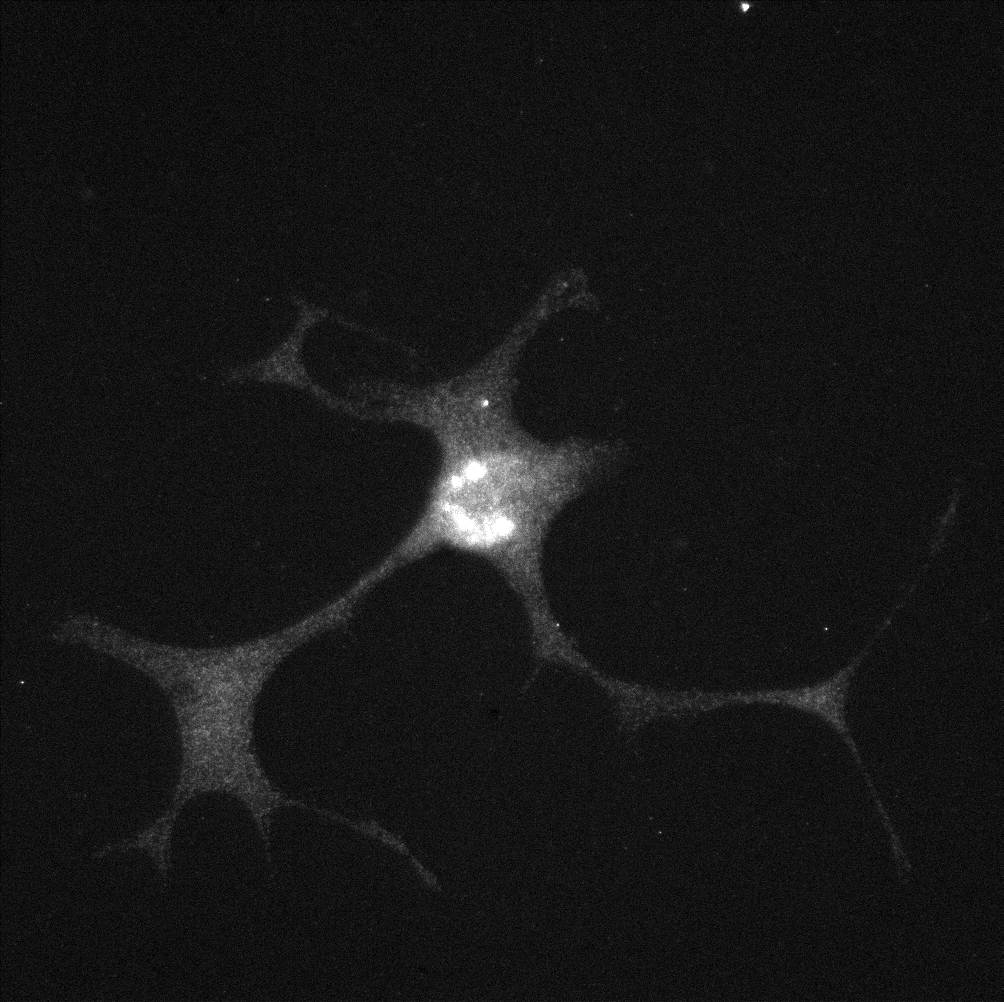

Supplement: Supplementary file 6 — Source data Fig. 5 [file 44319_2025_583_MOESM6_ESM.zip › Figure 6/6M/6M_FN_1-10_FAKi_pERK1-2.tif]

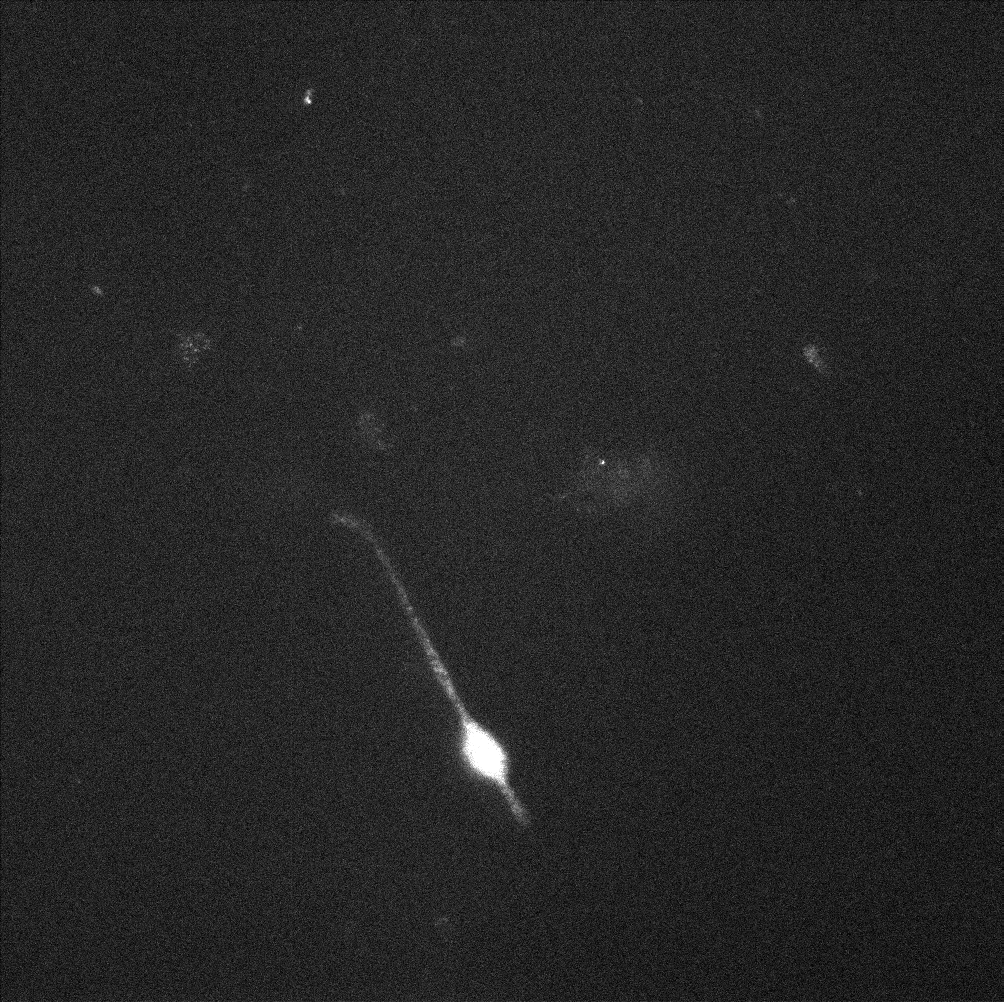

Supplement: Supplementary file 6 — Source data Fig. 5 [file 44319_2025_583_MOESM6_ESM.zip › Figure 6/6M/6M_COLI_1-10_FAKi_pERK1-2.tif]
