# Supplementary material for: Melanocyte differentiation and mechanosensation are differentially modulated by distinct extracellular matrix proteins
Source: EMBO Rep. 2025 Sep 19;26(21):5270–99. doi: 10.1038/s44319-025-00583-6 (PMC12592508; doi:10.1038/s44319-025-00583-6)
Supplement: Supplementary file 8 — Expanded View Figures [file 44319_2025_583_MOESM8_ESM.pdf]

Expanded View Figures

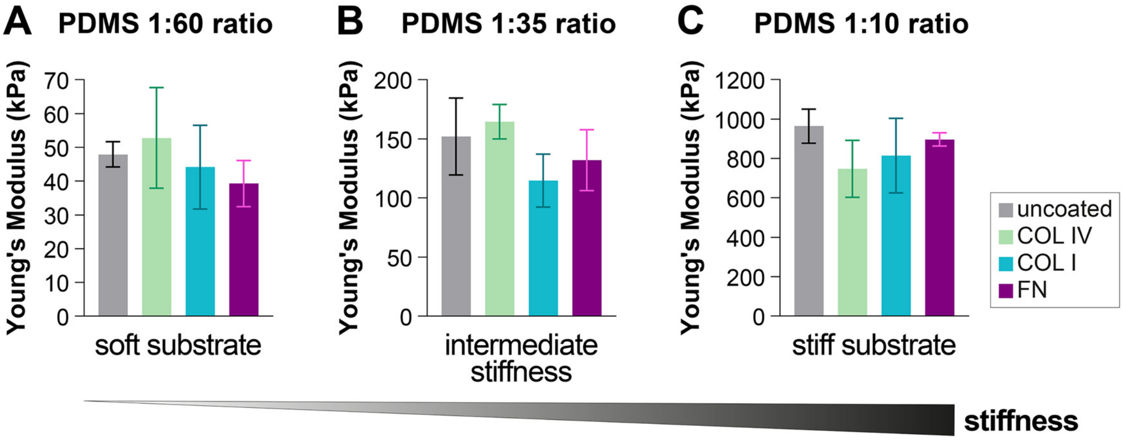

Figure EV1. Validation of PDMS elasticity using atomic force microscopy.

Young's modulus of PDMS substrates with varying crosslinker ratios. (**A**) PDMS substrate with a crosslinker:silicone base agent ratio of 1:60; means ± SD, $N = 3$ (biological replicates); Welch ANOVA tests: ns $p = 0.989$ (uncoated vs. COL I), ns $p = 0.4616$ (uncoated vs. FN), ns $p = 0.9842$ (uncoated vs. COL IV), ns $p = 0.982$ (COL I vs. FN), ns $p = 0.9505$ (COL I vs. COL IV), ns $p = 0.6723$ (FN vs. COL IV). (**B**) PDMS substrate with a 1:35 ratio; means ± SD, $N \geq 3$ (biological replicates); Welch ANOVA tests: ns $p = 0.2933$ (uncoated vs. COL I), ns $p = 0.8869$ (uncoated vs. FN), ns $p = 0.9686$ (uncoated vs. COL IV), ns $p = 0.9035$ (COL I vs. FN), ns $p = 0.0731$ (COL I vs. COL IV), ns $p = 0.5114$ (FN vs. COL IV). (**C**) PDMS substrate with a 1:10 ratio; means ± SD, $N = 3$ (biological replicates); Welch ANOVA tests: ns $p = 0.7579$ (uncoated vs. COL I), ns $p = 0.7492$ (uncoated vs. FN), ns $p = 0.3624$ (uncoated vs. COL IV), ns $p = 0.9483$ (COL I vs. FN), ns $p = 0.9939$ (COL I vs. COL IV), ns $p = 0.5694$ (FN vs. COL IV).

 © The Author(s)

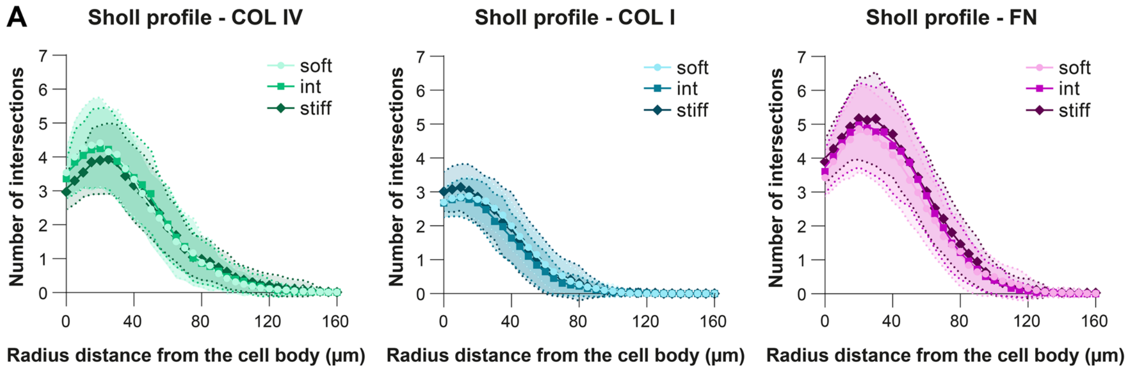

Figure EV2. Sholl profiles for iMCs exposed to different ECM molecules.

(**A**) Graphs showing the Sholl profile analysis, plotting the number of dendrite intersections against the distance from the cell body. The SEM are represented by the connecting curve (dotted line); $N = 3$ (biological replicates), n(cells) ≥85. Each curve represents a substrate stiffness condition, and each graph represents an ECM type (same cells as analyzed in Fig. 1C, different plotting of the data); Welch ANOVA test: COL IV: ns $p > 0.9999$ (1:60 vs. 1:35), ns $p = 0.9650$ (1:60 vs. 1:10), ns $p = 0.9489$ (1:35 vs. 1:10); COL I: ns $p = 0.5182$ (1:60 vs. 1:35), ns $p > 0.9999$ (1:60 vs. 1:10), ns $p = 0.5964$ (1:35 vs. 1:10); FN: ns $p = 0.8906$ (1:60 vs. 1:35), ns $p = 0.4342$ (1:60 vs. 1:10), ns $p = 0.7603$ (1:35 vs. 1:10).

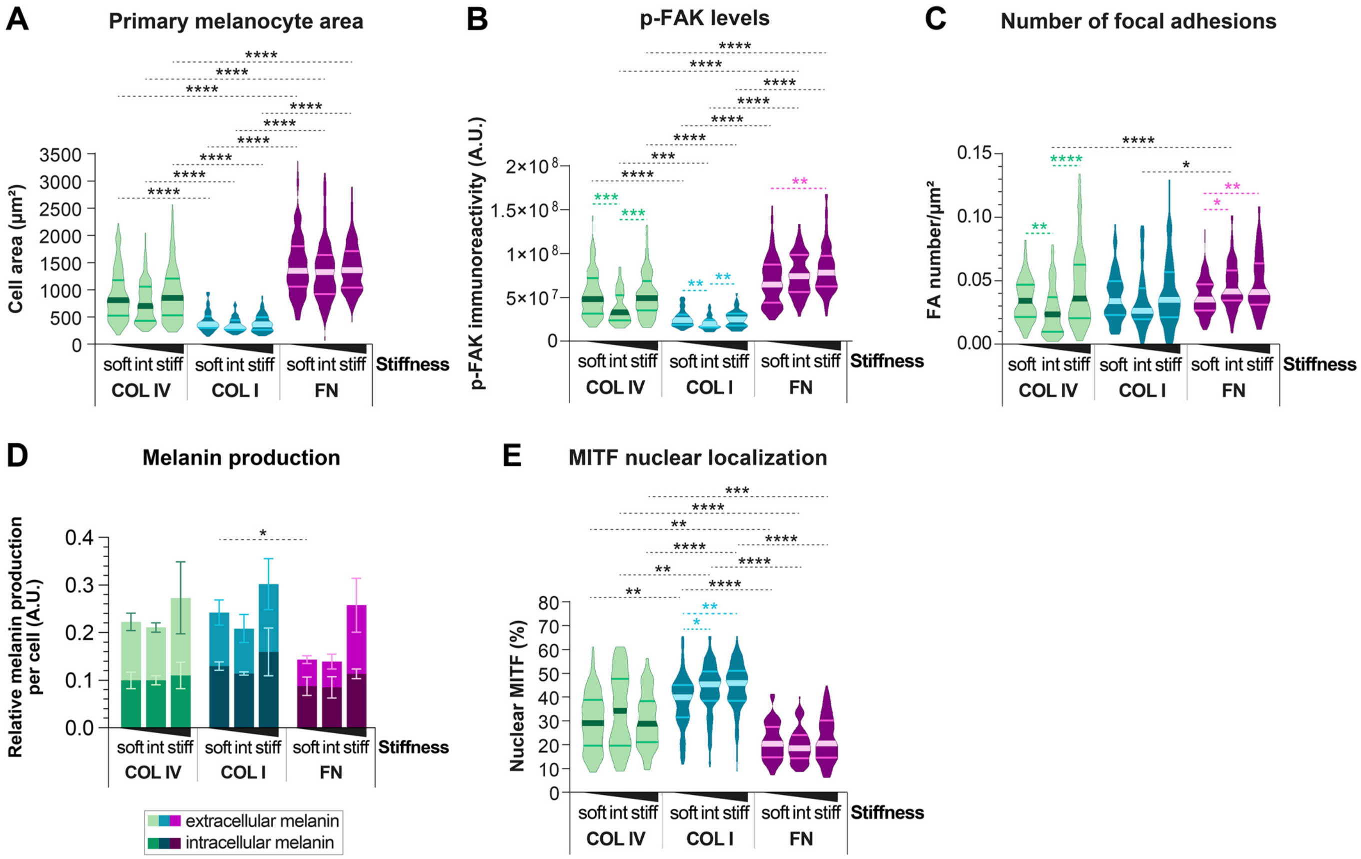

Figure EV3. Response of primary MCs to distinct ECM cues.

pMCs were cultured overnight on substrates of varying stiffness (soft, intermediate or stiff) coated with COL IV, COL I, or FN. (**A**) Violin plots showing the medians and distributions of the cell area of pMCs; $N \geq 5$ (biological replicates), n(cells) ≥96; Kruskal-Wallis test: COL IV: ns $p = 0.5372$ (1:60 vs. 1:35), ns $p > 0.9999$ (1:60 vs. 1:10), ns $p = 0.1807$ (1:35 vs. 1:10); COL I: ns $p = 0.1552$ (1:60 vs. 1:35), ns $p = 0.7629$ (1:60 vs. 1:10), ns $p > 0.9999$ (1:35 vs. 1:10); FN: ns $p = 0.4268$ (1:60 vs. 1:35), ns $p > 0.9999$ (1:60 vs. 1:10), ns $p = 0.0972$ (1:35 vs. 1:10); 1:60: ****$p < 0.0001$ (COL I vs. FN), ****$p < 0.0001$ (COL I vs. COL IV), ****$p < 0.0001$ (FN vs. COL IV); 1:35: ****$p < 0.0001$ (COL I vs. FN), ****$p < 0.0001$ (COL I vs. COL IV), ****$p < 0.0001$ (FN vs. COL IV); 1:10: ****$p < 0.0001$ (COL I vs. FN), ****$p < 0.0001$ (COL I vs. COL IV), ****$p < 0.0001$ (FN vs. COL IV). (**B**) Quantification of p-FAK levels; violin plots showing the medians and distributions of the integrated density of total p-FAK per cell; $N = 3$ (biological replicates), n(cells) ≥52; Kruskal-Wallis test: COL IV: ***$p = 0.0004$ (1:60 vs. 1:35), ns $p > 0.9999$ (1:60 vs. 1:10), ***$p = 0.0005$ (1:35 vs. 1:10); COL I: **$p = 0.002$ (1:60 vs. 1:35), ns $p > 0.9999$ (1:60 vs. 1:10), **$p = 0.0024$ (1:35 vs. 1:10); FN: ns $p = 0.3084$ (1:60 vs. 1:35), **$p = 0.0035$ (1:60 vs. 1:10), ns $p = 0.7979$ (1:35 vs. 1:10); 1:60: ****$p < 0.0001$ (COL I vs. FN), ****$p < 0.0001$ (COL I vs. COL IV), ns $p = 0.1$ (FN vs. COL IV); 1:35: ****$p < 0.0001$ (COL I vs. FN), ***$p = 0.0003$ (COL I vs. COL IV), ****$p < 0.0001$ (FN vs. COL IV); 1:10: ****$p < 0.0001$ (COL I vs. FN), ****$p < 0.0001$ (COL I vs. COL IV), ****$p < 0.0001$ (FN vs. COL IV). (**C**) Quantification of FAs; violin plots showing the medians and distributions of the number of focal adhesions per µm^2 per cell; $N = 3$ (biological replicates), n(cells) ≥65; Kruskal-Wallis test: COL IV: **$p = 0.0081$ (1:60 vs. 1:35), ns $p = 0.507$ (1:60 vs. 1:10), ****$p < 0.0001$ (1:35 vs. 1:10); COL I: ns $p = 0.7166$ (1:60 vs. 1:35), ns $p > 0.9999$ (1:60 vs. 1:10), ns $p = 0.1991$ (1:35 vs. 1:10); FN: *$p = 0.0117$ (1:60 vs. 1:35), **$p = 0.0071$ (1:60 vs. 1:10), ns $p > 0.9999$ (1:35 vs. 1:10); 1:60: ns $p > 0.9999$ (COL I vs. FN), ns $p > 0.9999$ (COL I vs. COL IV), ns $p > 0.9999$ (FN vs. COL IV); 1:35: *$p = 0.0167$ (COL I vs. FN), ns $p = 0.5377$ (COL I vs. COL IV), ****$p < 0.0001$ (FN vs. COL IV); 1:10: ns $p > 0.9999$ (COL I vs. FN), ns $p > 0.9999$ (COL I vs. COL IV), ns $p > 0.9999$ (FN vs. COL IV). (**D**) Quantification of intra- and extracellular melanin content by spectrophotometry at 405 nm from pMCs cultured 72 h on substrates of varying stiffness (soft, intermediate or stiff) coated with COL IV, COL I or FN; means ± SD, $N = 3$ (biological replicates); multiple t- tests: Soft vs. Intermediate: ns $p = 0.4947$ (COL I), ns $p = 0.8479$ (FN), ns $p = 0.8479$ (COL IV); Soft vs. Stiff: ns $p = 0.619892$ (COL I), ns $p = 0.10379$ (FN), ns $p = 0.619892$ (COL IV); Intermediate vs. Stiff: ns $p = 0.361807$ (COL I), ns $p = 0.118759$ (FN), ns $p = 0.361807$ (COL IV); COL I vs. COL IV: ns $p = 0.868243$ (1:60), ns $p = 0.935696$ (1:35), ns $p = 0.935696$ (1:10), COL IV vs. FN: ns $p = 0.053003$ (1:60), ns $p = 0.053003$ (1:35), ns $p = 0.836335$ (1:10); COL I vs. FN: *$p = 0.0139$ (1:60), ns $p = 0.0797$ (1:35), ns $p = 0.5564$ (1:10). (**E**) Quantification of nuclear MITF; violin plots showing the medians and distributions of the percentage of nuclear MITF per cell; $N = 3$ (biological replicates), n(cells) ≥67; Kruskal-Wallis test: COL IV: ns $p = 0.2081$ (1:60 vs. 1:35), ns $p > 0.9999$ (1:60 vs. 1:10), ns $p = 0.3965$ (1:35 vs. 1:10); COL I: *$p = 0.0186$ (1:60 vs. 1:35), **$p = 0.0034$ (1:60 vs. 1:10); ns $p > 0.9999$ (1:35 vs. 1:10); FN: ns $p = 0.7181$ (1:60 vs. 1:35), ns $p > 0.9999$ (1:60 vs. 1:10), ns $p = 0.2589$ (1:35 vs. 1:10); 1:60: ****$p < 0.0001$ (COL I vs. FN), **$p = 0.0056$ (COL I vs. COL IV), **$p = 0.0013$ (FN vs. COL IV); 1:35: ****$p < 0.0001$ (COL I vs. FN), **$p = 0.0046$ (COL I vs. COL IV), ****$p < 0.0001$ (FN vs. COL IV); 1:10: ****$p < 0.0001$ (COL I vs. FN), ****$p < 0.0001$ (COL I vs. COL IV), ***$p = 0.0009$ (FN vs. COL IV). AU, arbitrary units.

 © The Author(s)

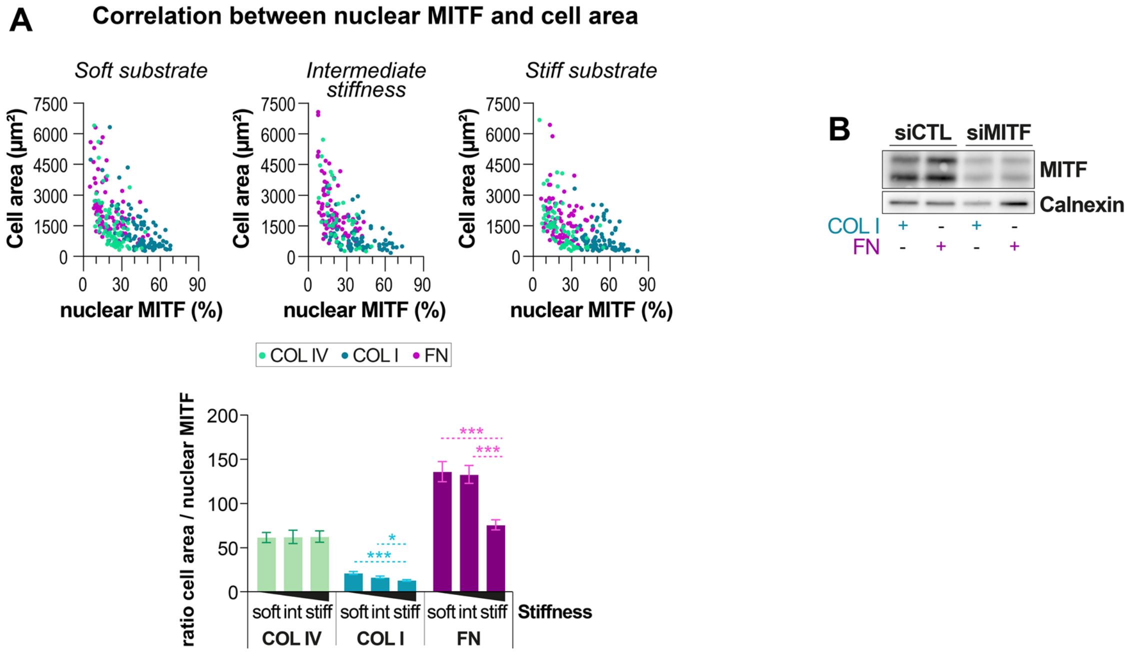

Figure EV4. Negative correlation between nuclear MITF levels and MC area and siMITF validation.

(**A**) The percentage of nuclear MITF was plotted against the cell area of the corresponding MC for all stiffnesses and ECM types tested. The bar diagram depicts the means ± SEM of the ratio of cell area to percentage of nuclear MITF per cell; $N = 3$ (biological replicates), n(cells) ≥47; Kruskal–Wallis test: COL IV: ns $p > 0.9999$ (1:60 vs. 1:35), ns $p = 0.5839$ (1:60 vs. 1:10), ns $p > 0.9999$ (1:35 vs. 1:10); COL I: ns $p = 0.5497$ (1:60 vs. 1:35), ***$p = 0.0001$ (1:60 vs. 1:10), *$p = 0.0329$ (1:35 vs. 1:10); FN: ns $p > 0.9999$ (1:60 vs. 1:35), ***$p = 0.0001$ (1:60 vs. 1:10), ***$p = 0.0001$ (1:35 vs. 1:10); 1:60: ****$p < 0.0001$ (COL I vs. FN), ****$p < 0.0001$ (COL I vs. COL IV), **$p = 0.0014$ (FN vs. COL IV); 1:35: ****$p < 0.0001$ (COL I vs. FN), ****$p < 0.0001$ (COL I vs. COL IV), ***$p = 0.0006$ (FN vs. COL IV); 1:10: ****$p < 0.0001$ (COL I vs. FN), ****$p < 0.0001$ (COL I vs. COL IV), ns $p = 0.0603$ (FN vs. COL IV). (**B**) Western blot analysis of MITF expression in iMCs plated on COL I or FN and transfected for 72 h with siCtrl or siMITF, performed in parallel with melanin quantification shown in Fig. 4D.

© The Author(s)

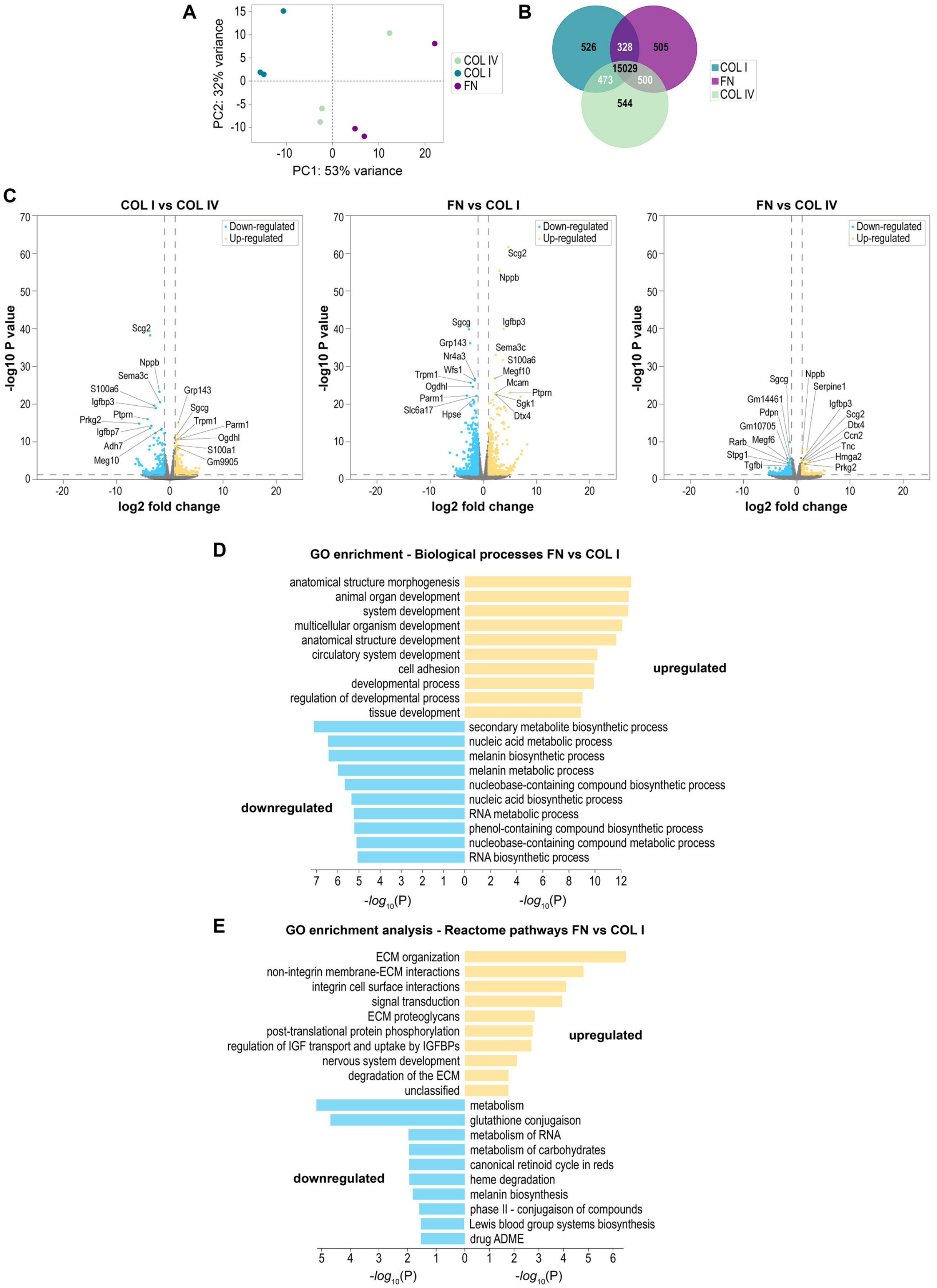
A
PC1: 53% variance
PC2: 32% variance
COL IV
COL I
FN
B
526
328
505
15029
473
500
544
COL I
FN
COL IV
C
COL I vs COL IV
FN vs COL I
FN vs COL IV
Down-regulated
Up-regulated
-log10 P value
log2 fold change
Scg2
Nppb
Sema3c
S100a6
Igfbp3
Ptprn
Prkg2
Igfbp7
Adh7
Meg10
Grp143
Sgcg
Trpm1
Parm1
Ogdhl
S100a1
Gm9905
Sgcg
Grp143
Nr4a3
Wfs1
Trpm1
Ogdhl
Parm1
Slc6a17
Hpse
Scg2
Nppb
Igfbp3
Sema3c
S100a6
Megf10
Mcam
Ptprn
Sgk1
Dtx4
Sgcg
Gm14461
Pdpn
Gm10705
Rarb
Megf6
Stpg1
Tgfbi
Nppb
Serpine1
Igfbp3
Scg2
Dtx4
Ccn2
Tnc
Hmga2
Prkg2
D
GO enrichment - Biological processes FN vs COL I
anatomical structure morphogenesis
animal organ development
system development
multicellular organism development
anatomical structure development
circulatory system development
cell adhesion
developmental process
regulation of developmental process
tissue development
upregulated
secondary metabolite biosynthetic process
nucleic acid metabolic process
melanin biosynthetic process
melanin metabolic process
nucleobase-containing compound biosynthetic process
nucleic acid biosynthetic process
RNA metabolic process
phenol-containing compound biosynthetic process
nucleobase-containing compound metabolic process
RNA biosynthetic process
downregulated
-log10(P)
E
GO enrichment analysis - Reactome pathways FN vs COL I
ECM organization
non-integrin membrane-ECM interactions
integrin cell surface interactions
signal transduction
ECM proteoglycans
post-translational protein phosphorylation
regulation of IGF transport and uptake by IGFBPs
nervous system development
degradation of the ECM
unclassified
upregulated
metabolism
glutathione conjugaison
metabolism of RNA
metabolism of carbohydrates
canonical retinoid cycle in reds
heme degradation
melanin biosynthesis
phase II - conjugaison of compounds
Lewis blood group systems biosynthesis
drug ADME
downregulated
-log10(P)

 © The Author(s)

Figure EV5. Transcriptomic profiling across ECM conditions.

iMCs were cultured overnight on stiff substrates coated with COL IV, COL I, or FN; $N = 3$ (biological replicates). (**A**) Principal component analysis (PCA) of gene expression across substrates. Each dot represents one sample: COL IV (green), COL I (blue), and FN (purple). Axes indicate principal components capturing the highest variance. (**B**) Venn diagram showing the number of genes expressed in the samples grown on COL IV, COL I, and FN substrates. (**C**) Volcano plots depicting differentially expressed genes between substrates: (i) COL I vs. COL IV, (ii) FN vs. COL I, and (iii) FN vs. COL IV (*p* values derived from Wald tests in DESeq2 with Benjamini–Hochberg adjustment). Significantly downregulated genes are shown in blue; upregulated genes in yellow. (**D**) Gene Ontology (GO) Biological Process enrichment analysis of significantly regulated genes across substrates. The x-axis indicates the adjusted *p* value of Fisher's Exact test; the y-axis lists the most significantly enriched biological processes. (**E**) Reactome pathway enrichment analysis performed on differentially expressed genes across ECM conditions. The x-axis represents the adjusted *p* value of Fisher's exact test; the y-axis lists the top significantly enriched Reactome pathways.

© The Author(s)

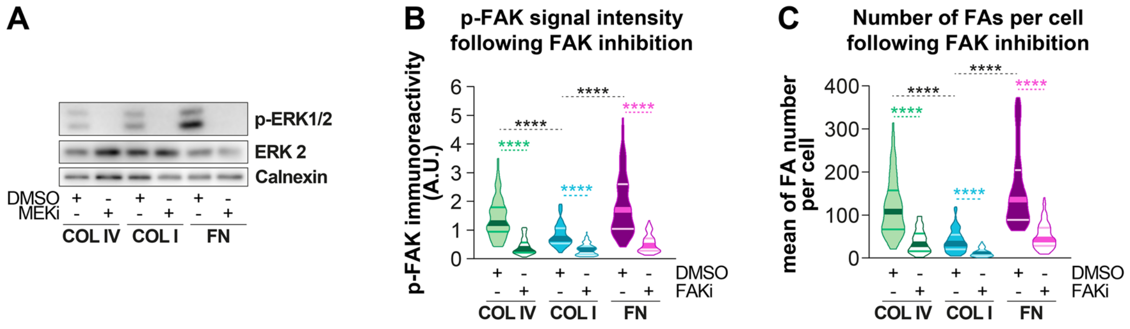

Figure EV6. Functional validation of MEK and FAK inhibitors in iMCs cultured on ECM-coated substrates.

iMCs were cultured on stiff substrates coated with COL IV, COL I, or FN. Four hours post-plating, a 16-h treatment with DMSO, 100 nM of MEK inhibitor (MEKi = Trametinib) or FAK inhibitor (FAKi = Ifebemtinib) was commenced. (**A**) Representative Western blot showing the inhibition of ERK1/2 phosphorylation with Trametinib treatment. (**B**) Quantification of p-FAK (Y397) intensity per cell following treatment with DMSO or FAK inhibitor (FAKi); violin plots display medians and distributions; $N = 3$ (biological replicates), n(cells) ≥88; Kruskal–Wallis test: DMSO: ****$p < 0{,}0001$ (COL IV vs. COL I), ns $p > 0{,}9999$ (COL IV vs. FN), **** $p < 0{,}0001$ (COL I vs. FN); DMSO vs. FAKi: **** $p < 0{,}0001$ (COL IV DMSO vs. COL IV FAKi 24 h), ****$p < 0{,}0001$ (COL I DMSO vs. COL I FAKi 24 h), ****$p < 0{,}0001$ (FN DMSO vs. FN FAKi 24 h). (**C**) Quantification of focal adhesion (FA) number per cell following FAKi treatment; violin plots show medians and distributions; $N = 3$ (biological replicates), n(cells) ≥70; Kruskal–Wallis test: DMSO: ****$p < 0.0001$ (COL IV vs. COL I), ns $p = 0.6525$ (COL IV vs. FN), ****$p < 0.0001$ (COL I vs. FN); DMSO vs. FAKi: ****$p < 0.0001$ (COL IV DMSO vs. COL IV FAKi), ****$p < 0.0001$ (COL I DMSO vs. COL I FAKi), ****$p < 0.0001$ (FN DMSO vs. FN FAKi).

 © The Author(s)
